# Targeted Fluoxetine Delivery Using Folic Acid-Modified PLGA Nanoparticles for Selective Uptake by Glioblastoma Cells

**DOI:** 10.3390/pharmaceutics17091116

**Published:** 2025-08-27

**Authors:** Maria João Ramalho, Carina Nóbrega, Stéphanie Andrade, Jorge Lima, Joana Angélica Loureiro, Maria Carmo Pereira

**Affiliations:** 1 LEPABE—Laboratory for Process Engineering, Environment, Biotechnology and Energy, Faculty of Engineering, University of Porto, 4200-465 Porto, Portugal; 2 ALiCE—Associate Laboratory in Chemical Engineering, Faculty of Engineering, University of Porto, Rua Dr. Roberto Frias, 4200-465 Porto, Portugal; 3 I3S—Instituto de Investigação e Inovação em Saúde, Universidade do Porto, R. Alfredo Allen, 4200-135 Porto, Portugal; 4 Ipatimup—Instituto de Patologia e Imunologia Molecular da Universidade do Porto, Rua Júlio Amaral de Carvalho 45, 4200-135 Porto, Portugal; 5 Faculty of Medicine, Porto University, 4200-319 Porto, Portugal; 6 Department of Mechanical Engineering, Faculty of Engineering, University of Porto, 4200-465 Porto, Portugal

**Keywords:** brain delivery, brain tumor, folate receptor, drug resistance, MGMT-mediated resistance, non-alkylating drug

## Abstract

**Background/Objectives**: The conventional treatment of glioblastoma (GBM) with alkylating agents is not curative. The protein O6-methylguanine DNA methyltransferase (MGMT) is a significant limitation, being able to repair drug-induced DNA damage. Thus, exploring non-alkylating agents already approved by the FDA is imperative. The antidepressant fluoxetine (FL) has been explored due to its anti-cancer properties. However, its first-pass effect and its non-targeted distribution to brain tissue are major limitations of FL’s administration, which is conventionally orally administered. Thus, the primary objective of this work was the development of poly(lactic-co-glycolic acid) (PLGA) nanoparticles (NPs) tailored with folic acid (FA) for FL delivery to GBM cells. **Methods**: A Central Composite Design (CCD) was applied to optimize the NPs. **Results**: The developed FA-functionalized PLGA NPs exhibited physicochemical properties suitable for brain-targeted delivery. The final formulation presented an average diameter of 167 ± 8 nm, a polydispersity index (PdI) of 0.23 ± 0.07, and a zeta potential of −22.2 ± 0.3 mV. The encapsulation efficiency (EE) and loading capacity (LC) values were 44.4 ± 3.8% and 3.1 ± 0.3%, respectively. In vitro studies demonstrated that the NPs are stable in storage and simulated physiological conditions and can maintain a controlled and slow-release profile of FL for 17 days. In vitro cell uptake experiments demonstrated that conjugation with FA enhances the NPs’ internalization in GBM cells overexpressing folate receptors through endocytosis mediated by this receptor. Furthermore, in vitro cytotoxicity experiments demonstrated that the FL encapsulation in the developed NPs maintains drug efficacy, as well as it was able to increase cell sensitivity to treatment with an alkylating agent. **Conclusions**: These results suggest that the developed NPs are effective nanocarriers, either as a standalone therapy or as a chemosensitizer in combination with the standard GBM treatment.

## 1. Introduction

Glioblastoma (GBM) is the most lethal and prevalent brain tumor in adults, corresponding to 48.6% of all central nervous system (CNS) tumors [1]. Its current treatment consists of surgery followed by a combination of chemotherapy and radiotherapy, which often leads to severe adverse effects and insufficient elimination of the tumor tissue [2]. This is reflected in high mortality rates, with patients’ survival between 12 and 15 months. Additionally, long-term survival rates are notably low, with only 5% of patients surviving beyond five years. Instances of survival exceeding five years are infrequent, highlighting the aggressive nature of this malignancy [3].

The alkylating agent temozolomide (TMZ) is the gold-standard drug for GBM’s chemotherapy. However, it possesses several limitations, including significant side effects, such as anemia, thrombocytopenia, leukopenia, and increased susceptibility to infections. Additionally, high resistance to therapy due to the action of the O6-methylguanine-DNA methyltransferase protein (MGMT) is one of the major limitations of TMZ and other alkylating agents. MGMT is a DNA repair protein able to protect cells from the impacts of alkylating agents by averting G:C→A:T genetic mutations, therefore neutralizing the cytotoxic effects of chemotherapy [4].

Thus, the methylation (silencing) of the MGMT gene promoter is a crucial prognostic factor in GBM [5]. Statistically, approximately 50% of GBM patients have an unmethylated MGMT promoter, leading to overexpression of the MGMT protein [6]. This overexpression is associated with a poor response to therapy involving alkylating agents, leading to a worse prognosis and highlighting the urgent need for effective new treatments. Recent large-cohort studies have shown that MGMT-methylated patients undergoing standard chemoradiation have a median overall survival of approximately 16.4 months, compared to 11.8 months in unmethylated patients, confirming the strong prognostic value of MGMT status in clinical outcomes [7].

Therefore, repurposing FDA-approved non-alkylating drugs for the treatment of GBM has become a valuable strategy [8]. This approach presents the possibility of accelerating the creation of novel therapeutic alternatives to treat this devastating disease. In fact, different agents have been explored in clinical trials for repurposing in GBM to their recently discovered anti-cancer activity, such as metformin [9], disulfiram [10], ritonavir [11], and fluoxetine (FL).

FL is a selective serotonin reuptake inhibitor (SSRI) used in different psychiatric disorders [12], such as bipolar depression, obsessive-compulsive, panic disorder, and major depression [13]. It has been demonstrated that this drug has anti-cancer activity owing to its ability to inhibit the sphingomyelin phosphodiesterase 1 (SMPD1) enzyme. This enzyme is responsible for catalyzing the conversion of sphingomyelin to ceramide and is related to the overall survival in patients with GBM (a higher SMPD1 indicates a smaller survival). Therefore, when SMPD1 is inhibited, the level of sphingomyelin increases, and GBM cells are killed through inhibition of epidermal growth factor receptors, responsible for the growth and proliferation of cancer cells, and activation of lysosomal stress, leading to the loss of their integrity, endangering their viability [14,15,16]. Several studies demonstrated FL’s efficacy for GBM therapy in in vitro and in vivo models [17,18,19].

However, the hepatic metabolism of FL when administered orally reduces drug bioavailability to below 90%, limiting its efficacy. Additionally, its lipophilic nature leads to non-specific accumulation in various tissues, rather than being targeted specifically to the brain. FL exhibits a moderate brain-to-plasma ratio, whereas other SSRIs demonstrate higher values [20].

Nanoparticles (NPs) for FL-targeted delivery are a proper strategy to ensure specificity for the brain tumor tissue, preventing drug accumulation in other organs and thus enhancing their anti-GBM therapeutic efficacy. NPs tend to selectively accumulate in tumor tissues by passive targeting mainly due to the *Enhanced Permeability and Retention* (EPR) effect verified in fast-growing solid tumors, like GBM [21]. Other advantages of nanoencapsulation are the ability to improve drug half-life and to protect the encapsulated drug from degradation, which leads to better stability and continued drug release [22]. However, drug delivery to brain tumor tissues faces an additional obstacle, the need to cross the blood–brain barrier (BBB). The most promising approach to promote BBB crossing is to use active targeting strategies by modifying the surface of the NPs with ligands with affinity for the receptors overexpressed in the BBB cells. Folic acid (FA) is one of the most popular targeting ligands for GBM delivery, due to an overexpression of folate receptors not only in the BBB’s endothelial cells [23] but also in the GBM cells [24]. This is evidenced by the many FA-conjugated NPs proposed in recent years for delivering various drugs in GBM therapy [25,26].

Thus, this work was intended to develop poly(lactic-co-glycolic acid) (PLGA) NPs conjugated with FA for the delivery of FL to GBM cells. PLGA is a copolymer of poly-lactic acid and poly-glycolic acid commonly used in biomedical applications due to its biosafety, biocompatibility, and biodegradability [27]. While FL-loaded NPs have shown promise in studies for other diseases [28,29,30,31], their application specifically for GBM treatment has not yet been explored. Moreover, to the best of our knowledge, FA-conjugated PLGA NPs have not been reported for FL encapsulation in any disease context.

The FL-loaded NPs production was optimized using a central composite design (CCD) and functionalized with FA to enhance specificity for brain tumors. The optimized NPs were then thoroughly characterized in vitro for their physicochemical properties, morphology, stability, and release profile. Cellular assays were performed using GBM cells (U251, U87, and T98G) and healthy astrocytes (NHA) as controls to assess cellular internalization and the role of FA in receptor-mediated endocytosis via folate receptors. Finally, the antiproliferative activity of the NPs and their ability to sensitize cells to the effects of the standard therapeutic agent for GBM—temozolomide (TMZ)—were evaluated.

## 2. Materials and Methods

### 2.1. Materials

FL hydrochloride was purchased from the British Pharmacopoeia (London, UK). PLGA (100%, 50:50; MW 5000–10,000) was purchased from Thermo Scientific (Hillsboro, OR, USA). Polyvinyl alcohol (PVA, Mowiol^®^ 4–88, ≥99.5%, MW 31,000), N-(3-dimethylaminopropyl)-N′-ethylcarbodiimide hydrochloride (EDC, ≥99.8, MW 192), FA (≥97%, MW 441), phosphate-buffered saline (PBS), acetic acid (≥98%, MW 60), coumarin-6 (C6, 98%, MW 350), trichloroacetic acid (TCA) (99%, MW 163), tris(hydroxymethyl)aminomethane (≥99.8%, MW 141), sulforhodamine B (SRB) (MW 581), sodium hydroxide (NaOH, ≥97%, MW 40.0), and polyoxyethylene (10) isooctylcyclohexyl ether (Triton X-100) were acquired from Sigma Aldrich (St. Louis, MO, USA). Ethanol (99%, MW 46) was obtained from Avantor (Radnor, PA, USA). Ethyl acetate (99.6%, ACS reagent, MW 88) was acquired from BioVision (Waltham, MA, USA). Dimethyl sulfoxide (DMSO) (≥99.9, MW 78) was acquired from VWR International (Radnor, PA, USA). Uranyl acetate (99.6%) was purchased from Electron Microscope Sciences (Hatfield, PA, USA). Fetal bovine serum (Gibco^TM^ FBS) and trypsin (Gibco^TM^ TrypLE^TM^) were purchased from Fisher Scientific (Hampton, NH, USA). Trypan blue (≥70%, MW 961) was acquired from Biochem Chemopharma (Cosne-Cours-sur-Loire, France). Penicillin-streptomycin solution (×100) and funzigone were purchased from Biowest LCC (Riverside, MO, USA). High-glucose Dulbecco’s Modified Eagle medium (DMEM) was bought from Capricorn Scientific (Ebsdorfergrund, Germany).

### 2.2. Cells

T98G, U251, and U87MG human GBM cell lines and immortalized human astrocytes (NHA) were obtained from the American Type Culture Collection (ATCC^®^, Manassas, VA, USA). Cell culture was performed in DMEM, supplemented with 10% (*v*/*v*) FBS, 1% (*v*/*v*) penicillin-streptomycin, and 0.5% (*v*/*v*) fungizone. The cells were passaged twice a week and maintained in a humidified incubator at 37 °C with 5% CO_2_.

### 2.3. Optimization of the Protocol for FL-Loaded PLGA NPs Preparation

#### 2.3.1. Preparation of FL-Loaded PLGA NPs

The double emulsion-evaporation of the solvent method was employed for the preparation of the FL-loaded PLGA NPs. Initially, a known amount of FL was dissolved in ethanol (0.20 mL) and mixed with 0.30 mL of ultrapure water. Subsequently, a solution of a known amount of PLGA in ethyl acetate was prepared (1.0 mL). FL and PLGA quantities varied in the different formulations as detailed in Table S1. The aqueous solution containing FL was mixed with the organic phase containing PLGA, and the mixture was subjected to a vortex for 30 s to prepare a water-in-oil emulsion. Afterward, the sample was emulsified by sonication at an amplitude of 70% (UP400S ultrasonic processor, Hielscher, Berlin, Germany). The duration of the sonication cycles varied in the different formulations as detailed in Table S1. Then, a water-in-oil-in-water emulsion was prepared by adding 1.0 mL of an aqueous solution of a known amount of PVA to the previously prepared water-in-oil emulsion. The PVA concentration varied in the different formulations as detailed in Table S1. Afterward, the sample was vortexed and emulsified as described above. After the sonication, the sample was quickly transferred to a beaker and placed in a magnetic stirrer plate (800 rotations per minute/rpm, Multistirrer 15, VWR International, Radnor, PA, USA) to promote the complete evaporation of the solvent.

To collect the NPs, a sequence of centrifugation steps was performed, by increasing the speed from 12,500 to 14,500 rpm and from 2 to 30 min (MiniSpin^®^plus, Eppendorf, Germany). The obtained pellet was resuspended in ultrapure water and saved for further NPs’ physicochemical characterization. To make sure that all excess PLGA and PVA were removed, the obtained supernatants were filtered using an Amicon filter (Amicon^®^ Ultra 0.5 mL Centrifugal Filter Devices, 3 kDa, Merck, Darmstadt, Germany). The filtered supernatants were saved for further non-entrapped drug encapsulation.

#### 2.3.2. Central Composite Design

The NPs protocol production was optimized by implementing a CCD. The CCD model is one of the most frequently used experimental designs, and it is extensively implemented for experimental response surface optimization since it is based on a fractional factorial design with center points (0) and an additional set of axial points (α), enabling the estimation of curvature within the model. As a result, this second-order model examines factors at five distinct levels (−α, −1, 0, +1, +α) [32]. Here, level −1 (low) corresponds to the minimum value of the parameter under investigation, while level +1 (high) represents its maximum value.

The chosen experimental factors were the amount of PLGA (mg) and fluoxetine (mg), the concentration of PVA (% *w*/*v*), and the time of sonication (s) (Table 1). The remaining parameters were maintained constant, such as the number of sonication cycles (2), the volume of the solvents (0.5 mL of inner aqueous phase, 1.0 mL of organic solvent, and 2.0 mL of outer aqueous phase), and others. The studied responses were the mean diameter of the NPs, the polydispersity index (PdI), the zeta potential, the loading capacity (LC), and the encapsulation efficiency (EE).

The CCD model was implemented using Design Expert software (version 13.0.5.0, Stat-Ease Inc., Minneapolis, MN, USA). Varying the independent variables within the previously defined experimental levels, the software generated a plan of 27 independent formulations (with 3 replicates of the center points). In Table S1, the different formulations and their respective response variables are presented.

Each response variable was individually fitted to a polynomial regression model, determining the effect of each experimental variable and the studied responses, using the following equation below:

(1) $$Y=\;b_{0}+b_{1}X_{1}+\cdots +b_{k}X_{k}+b_{12}X_{1}X_{2}+b_{13}X_{1}X_{3}+\cdots +b_{k-1,k}X_{k-1}X_{k}\;+b_{11}X_{1}^{2}+\cdots +b_{kk}X_{k}^{2}+\epsilon$$

The constants represent the following: *Y* the dependent response variable, *X_k_* the independent variable, *b*_0_ intercept, *b_k_* coefficients of the regression model, *K* the number of independent variables, and *ϵ* the error term. ANOVA statistical analysis was conducted, and *p*-values less than 0.05 were considered significant at a 95% confidence level.

### 2.4. Functionalization of FL-Loaded PLGA NPs with FA

The surface of the developed FL-loaded PLGA NPs was tailored with FA through a carbodiimide coupling reaction. Firstly, the NPs were incubated with EDC at a molar excess of 100× for 30 min at room temperature with agitation. EDC was used to activate the carboxyl groups of PLGA, allowing for further conjugation with the primary amine group of FA, by creating an amide bond between the PLGA and the FA [33]. Following this, the NPs with activated carboxylic acids were incubated with FA for 1 h at room temperature at 200× molar excess. After incubation, the NPs were collected by centrifugation as described in Section 2.3.1 and resuspended in ultrapure water. The choice of EDC:PLGA and FA:PLGA ratios were based on preliminary experiments. This excess was used to ensure efficient activation of the carboxyl groups for subsequent conjugation under mild reaction conditions.

Attenuated total reflectance (ATR) Fourier-transform infrared spectroscopy (FTIR) was further used to confirm the presence of FA on the surface of the NPs. A Bruker Alpha-P FT-IR Spectrometer (Bruker Optics Inc., Billerica, MA, USA) and Opus Software (version 6.5, Bruker Optik GmbH, Ettlingen, Germany) were used to record the FTIR spectrum of the produced FA-FL-loaded PLGA NPs. As a control, the spectra of non-conjugated FL-loaded NPs, free FL, PLGA polymer, and FA stock solution were also measured. For each sample, a 10 μL drop was directly placed into the ATR crystal and then dried with a nitrogen flow. The spectra were captured in absorbance mode with 64 scans for each sample and background observations and a wavenumber range of 4000–375 cm^−1^. The spectra analysis was performed in GraphPad Prism (version 9.1.2, GraphPad Software, Boston, MA, USA).

### 2.5. Physicochemical Characterization of NPs

The NPs’ characterization was conducted through the analysis of the mean size, the PdI, and the zeta potential by Dynamic Light Scattering coupled with Laser Doppler Electrophoresis (ZetaSizer Nano ZS equipment, Malvern Instruments, Malvern, UK). For the measurements, the NPs were diluted in ultrapure water to a final concentration of NPs of 3 mg/mL. To determine the mean size and PdI, a polystyrene standard cell (Sarstedt^®^) was used. To measure the zeta potential, a disposable folded capillary cell (DTS1070) was used. For all measurements, the refractive index of water as the dispersant at 25 °C was set to 1.330, the viscosity to 0.8872 cP, and the dielectric constant to 78.2. The polymer refractive index was fixed at 1.590, with an absorption value of 0.010. All obtained data were analyzed using the ZetaSizer software (version 8, Malvern, UK).

The colloidal stability of the FA-conjugated and non-conjugated FL-loaded PLGA NPs in storage conditions was evaluated in terms of variations in the mean size, PdI, and zeta potential. For that, the NPs were stored in aqueous suspension at 4 °C, and DLS measurements were conducted once a week over 10 weeks (final concentration of 3 mg/mL).

Morphological analysis was conducted by transmission electron microscopy (TEM). Briefly, 10 μL of the NPs’ suspension diluted in water (final concentration of 3 mg/mL) was placed on a copper grid (Formvar/Carbon—400 mesh Copper, Agar Scientific, Rotherham, UK) for 5 min and then stained with 2% (*w*/*v*) uranyl for 45 s. Then, the samples were air-dried for visualization using a Jeol JEM 1400 electron microscope at an accelerating voltage of 80 kV (Tokyo, Japan).

### 2.6. Determination of the FL Encapsulation Efficiency and NPs’ Loading Capacity

The EE of FL and NPs’ LC were determined indirectly by quantifying the non-encapsulated drug present in the supernatant. The supernatant was obtained after centrifugation and filtering with Amicon filters as described in Section 2.3.1. The free FL in the supernatant was quantified by the UV–Vis absorbance at a maximum wavelength of 263 nm using a microplate reader (Synergy 2 Microplate Reader, BioTek, Swindon, UK). The obtained absorbance values were correlated to a FL calibration curve in 5.0% (*w*/*v*) PVA. Then, the EE and LC values were determined using the following equations:

EE (%) = entrapped drug (mg)/total amount of used drug (mg) × 100(2)

LC (%) = entrapped drug (mg)/total amount PLGA (mg) × 100(3)

### 2.7. In Vitro Release of FL from FA-Conjugated and Non-Conjugated PLGA NPs

The amount of FL released from the non-conjugated and FA-conjugated PLGA NPs was determined using dialysis membranes (cellulose membrane, 6–8 kDa, SpectrumLabs, San Francisco, CA, USA) and employing a well-established in vitro static digestion model that chronologically mimics the different stages of digestion, including the stomach and small intestine [34]. For that, 118 mg of NPs (corresponding to 3.66 mg of entrapped FL) were dispersed in different biorelevant media (FaSSGF, pH 1.2; FaSSIF, pH 6.5; and PBS, pH 7.4). First, the NPs were placed for 2 h in FaSSGF (pH 1.2) at 37 °C with mild agitation (100 rpm) to simulate the movement of gastrointestinal fluids in vivo. Samples were taken every 15 min for the quantification of released FL (at 0, 15, 30, 45, 60, 75, 90, and 120 min). Next, the NPs were transferred to FaSSIF at 37 °C with gentle agitation (100 rpm) for 3 h, with aliquots collected every 15 min (at 135, 150, 165, 180, 195, and 210 min). Afterward, the NPs were incubated in PBS (0.01 M, pH 7.4) to simulate blood circulation conditions until the end of the experiment (17 days), with samples taken at pre-determined time points (3 h 15 min, 3 h 30 min, 3 h 45 min, 4 h, 4 h 30 min, 5 h, 5 h 15 min, 5 h 30 min, 5 h 45 min, 6 h, 6 h 15 min, 6 h 30 min, 6 h 45 min, 7 h, 7 h 30 min, 24 h, 48 h, 72 h, 144 h (6 days), 216 h (9 days), 240 h (10 days), 312 h (13 days), 360 h (15 days), and 408 h (17 days). At each time point, the released FL was quantified by UV–vis spectroscopy absorbance measurements at a maximum wavelength of 263 nm (Synergy 2 Microplate Reader, BioTek, Swindon, UK) and correlated to a FL calibration curve. A graph was then plotted exhibiting the percentage of FL released as a function of time.

To further characterize the drug release profile, the experimental data were fitted to different kinetic models, including Zero Order, First Order, Korsmeyer–Peppas, Higuchi, and Hixson–Crowell models. The kinetic parameters (*k*, *n*) and *R*^2^ were determined to select the best-fitting model and determine the major drug release mechanism. All mathematical fittings were performed using KinetDS (version 3.0, Jagiellonian University Medical College, Krakow, Poland).

The NPs’ stability in the simulated physiological conditions was evaluated for the duration of the release experiments by DLS measurements.

### 2.8. In Vitro Internalization Studies

#### 2.8.1. Quantitative Analysis by Fluorescence Measurements

The cellular uptake of both FA-conjugated and non-conjugated NPs was quantified by fluorescence measurements in GBM cells with high expression of the folate receptor (U251, U87, and T98G cells). As a control, NHA cells were used owing to their lower folate receptor expression. For the experiments, 8000 cells/well were seeded in 96-well plates (TPP^®^ tissue culture plates) and allowed to adhere for 24 h. Following this, FA-conjugated and non-conjugated PLGA NPs were diluted in DMEM to achieve a final polymer concentration of 20 μM (100 μL). To allow for NPs detection, coumarin-6 (C6) was loaded in the NPs by the single-emulsion evaporation technique. The cells were incubated with the NP internalization for two different periods (30 min and 120 min). At the end of the treatment, cells were washed with PBS to eliminate non-internalized NPs. The cells were incubated for 15 min at room temperature with 0.1% Triton X-100 in 0.1 M NaOH to promote their lysis for fluorescence quantification. The fluorescence of C6-NPs was measured at 430/485 nm excitation/emission wavelengths (Synergy 2 Microplate Reader, BioTek, Swindon, UK).

#### 2.8.2. Folate Receptor Blocking Experiment

To further investigate the role of the folate receptor in the uptake mechanism of the FA-conjugated NPs, a competitive receptor-blocking assay was conducted. Briefly, 8000 cells/well were seeded in 96-well plates and allowed to adhere for 24 h. Aiming to block the folate receptor prior to treatment with the NPs, the cells were first incubated with excess FA diluted in DMEM at concentrations ranging from 0.001 to 100 pM (100 μL) for 1 h. After the incubation period, the unbound FA was removed, and FA-conjugated and non-conjugated C6-PLGA NPs were added to the cells at a final concentration of 20 μM in DMEM (100 μL). Following a 2-h incubation period, the cells were processed for fluorescence measurements as described in the previous section.

### 2.9. In Vitro Cytotoxicity Studies

#### 2.9.1. Evaluation of the Antiproliferative Effect of the Developed NPs

The antiproliferative effect of non-conjugated and FA-conjugated FL-loaded PLGA NPs was evaluated in comparison to free FL across different human GBM cell lines (U251, U87, T98G) using the Sulforhodamine B (SRB) colorimetric assay. Healthy astrocyte cells (NHA) were also used as controls. Briefly, the cells were seeded in 96-well plates (TPP^®^ tissue culture plates) at a density of 1000 cells per well and allowed to adhere for 24 h in a humidified incubator (37 °C, 5% CO_2_). Following the adhesion period, cells were treated with different concentrations of free FL, FL-PLGA NPs, and FA-FL-PLGA NPs, prepared in DMEM medium at final FL concentrations ranging from 0.1 to 300 µM. As controls, unloaded PLGA and FA-PLGA NPs (at a polymer concentration ranging from 50 to 300 µg/mL) were included to determine any potential effects on cell growth of the used materials. Non-treated cells served as negative controls in all assays. After 72 h of incubation, cells were first fixed with 10% (*w*/*v*) TCA at 4 °C for 1 h and then stained with 0.4% (*w*/*v*) SRB dye for 30 min. Excess dye was removed by washing the cells with 1% (*v*/*v*) acetic acid, followed by air-drying. The protein-bound dye was then solubilized using 10 mM Tris buffer, and absorbance was measured at 560 nm (Synergy 2 Microplate Reader, BioTek, Swindon, UK). Each experimental condition was tested in triplicate, and the experiments were independently repeated three times. Cell proliferation was quantified by comparing the absorbance values of treated cells to those of untreated controls. The dose–response curves were obtained by plotting cell growth as a function of drug concentration. Then, the half-maximal inhibitory concentration (IC50) values for FL were obtained through non-linear regression of the dose–response curves using GraphPad Prism (version 9.1.2, GraphPad Software, San Diego, CA, USA).

#### 2.9.2. Evaluation of the Ability of FL-Loaded NPs in Sensitizing GBM Cells to TMZ

The ability of non-conjugated and FA-conjugated FL-loaded PLGA NPs to enhance cells’ sensitivity to the antineoplastic effects of the gold-standard alkylating drug used for GBM therapy (TMZ) was also evaluated using the SRB colorimetric assay. For this purpose, U251, U87, T98G, and NHA cells were seeded at a density of 1000 cells per well in 96-well culture plates (TPP^®^ tissue culture plates) and allowed to adhere for 24 h. Subsequently, the cells were treated with TMZ, diluted in DMEM to achieve final concentrations ranging from 0.1 to 3000 µM, both alone and in combination with non-conjugated or FA-conjugated FL-loaded PLGA NPs at a fixed FL concentration (15 µM for GBM cells and 25 µM for healthy astrocyte cells). The FL concentrations were selected based on the antiproliferative experiments described in Section 2.9.1, by employing the IC80 dose for each cell line. Non-treated cells served as negative controls. Following a 72-h incubation period, the SRB assay was conducted according to the protocol described in the previous section.

### 2.10. Statistical Analysis

Three independent experiments were conducted for each study, and for cell studies within each independent experiment, each condition was tested in triplicate. Statistical analysis was carried out using the Student’s *t*-test with a 95% confidence level. Differences were considered significant when the *p*-value was less than 0.05.

## 3. Results and Discussion

### 3.1. ANOVA Statistical Analysis of the CCD

A CCD was implemented to optimize the production of the FL-loaded PLGA NPs. Applying an experimental design offers several advantages over one-factor-at-a-time (OFAT) approaches that consist of trial-and-error, such as higher accuracy, reduction in experimental runs, and the ability to study the interactions between different factors [35]. Despite requiring fewer experiments, CCD ensures sufficient data robustness for reliable model prediction and response surface analysis. This makes it a widely adopted approach for the optimization of polymeric NPs [36,37] and other nanodelivery systems [38,39,40].

ANOVA statistical analysis was performed to evaluate how the selected independent variables (PLGA mass, fluoxetine mass, concentration of PVA, and sonication time) influence the studied dependent variables (size, PdI, zeta potential, EE, and LC). The regression model that best fitted each response was selected as follows: the quartic model for the size, the sixth-order model for the PdI, the cubic model for the zeta potential, the linear model for the EE, and the quadratic model for the LC, respectively. Table S1 in the supplementary file presents the results of the ANOVA statistical analysis for each response variable. From Table S1, it was concluded that all regression models were statistically significant (*p* < 0.05), except for the PdI, which, for this reason, was not included in the model.

Following the ANOVA analysis, regression coefficients (representing the relationship between the independent and dependent variables) were obtained, allowing us to derive equations to predict each response variable (Equations (S1)–(S4) in the supplementary information file). Additionally, contour plots (2D) and surface plots (3D) were obtained to visually illustrate the impact of each experimental variable on the studied responses (Figures S1–S4 in the Supplementary Information File).

#### 3.1.1. Analysis of Experimental Factors Affecting NPs’ Size

The size of the produced FL-loaded PLGA NPs varied between 136.7 (nanoformulation 10) and 379.3 nm (nanoformulation 15) (Table S1 in the Supplementary Information File). The obtained regression equation and the 2D and 3D plots (Figure S1 and Equation (S1) in the Supplementary Information File) showed that for PLGA amounts below 20 mg, the NPs’ size increases with the increase in PLGA amount. According to the literature, increasing the polymer concentration leads to NPs with larger sizes, due to an increased viscosity of the organic phase, which leads to a slower diffusion of the emulsion droplets into the aqueous phase [41]. However, for PLGA amounts above 20 mg, the opposite observation was verified, with the PLGA amount exerting a negative effect on the response. Other authors have reported this effect for higher PLGA concentrations, demonstrating that the NPs’ size might decrease due to a reduced Ostwald ripening. When the viscosity of the organic phase increases due to increased PLGA content, the diffusion rate decreases. This slower rate lowers the rate of the Ostwald ripening, resulting in the production of smaller NPs [42,43]. Therefore, the positive or negative effect of the PLGA amount on the size of the NPs is dependent on the concentration of the polymer.

Furthermore, it was verified that the effect of the FL amount on the NPs’ size is also dependent on its concentration. As the concentration of the drug increases, it may result in supersaturation, causing a faster nucleation rate, thereby forming smaller NPs. Nevertheless, high supersaturation can increase the odds of particle collision, leading to bigger NPs. In this way, it is expected that up to the threshold, the NP size decreases, followed by an increase in NP size beyond this point [44].

Additionally, the PVA concentration exerted a negative effect on the NP size due to the reduction in the interfacial tension between both phases (aqueous and organic), preventing the smaller NPs from coalescing into larger ones [41]. Interestingly, Equation (S1) also predicted that an increase in sonication time would result in a decrease in NP size. This is consistent with what has been observed by other authors who reported that longer sonication times lead to a faster and more uniform dispersion, thereby reducing NP size [45].

#### 3.1.2. Analysis of Experimental Factors Affecting NPs’ Zeta Potential

The zeta potential values of the produced FL-loaded PLGA NPs varied from −19.2 (nanoformulation 5) to −11.3 mV (nanoformulation 10) (Table S1 in the Supplementary Information File). As expected, all the obtained zeta potentials were negative due to the negatively charged terminal carboxyl groups of PLGA. From Figure S2 and Equation (S2) in the Supplementary Information File, it can be concluded that all independent variables exert a positive influence on this response, except for the concentration of PVA (Figure S2 and Equation (S2)). As PVA possesses negative ionized hydroxyl groups, increasing the PVA concentration led to making the zeta potential values more negative [46].

Concerning the sonication time, it was already reported by other authors that when the sonication time increases, it could induce the fragmentation of the NPs’ structure [47]. Moreover, the adsorption of FL on the PLGA NPs’ surface can mask the negative charge of the carboxylic groups of the PLGA, increasing the zeta potential values [48]. Furthermore, an increase in PLGA amount also leads to an increase in the zeta potential, thereby making the values less negative. This happens due to an increase in the coating layers on the NPs’ surface, forming a “shield” around the negative carboxyl groups, thus increasing the zeta potential [49].

#### 3.1.3. Analysis of Experimental Factors Affecting NPs’ EE

The EE values of the produced FL-loaded PLGA NPs varied from 8.3% (nanoformulation 5) to 58.4% (nanoformulation 6) (Table S1 in the Supplementary Information File). According to Figure S3 and Equation (S3) in the Supplementary Information File, all the independent variables positively influenced the EE of the NPs. At higher PLGA concentrations, the polymer precipitates quickly, slowing drug leakage from the NPs [50]. Furthermore, increasing the concentration of PVA led to an increase in the viscosity of the external aqueous phase, preventing the drug from diffusing from the internal aqueous phase to the outer aqueous phase. Moreover, higher PVA concentrations allowed better stabilization of the emulsion, which also enhanced drug entrapment [51]. Finally, it was also expected that an increase in sonication time would lead to an increase in the NPs’ EE. Ultrasound causes molecular vibrations, causing the polymer chains to disentangle. This process leads to a decrease in molecular weight of the polymer, causing dispersion of the drug and increasing the EE [52].

#### 3.1.4. Analysis of Experimental Factors Affecting NPs’ LC

The LC of the produced FL-loaded PLGA NPs ranged from 0.06% (nanoformulation 5) to 8.66% (nanoformulation 10). Analysis of Figure S4 and Equation (S4) (Supplementary File) indicates that an increase in the mass of PLGA has a negative impact on LC values, as these two variables are inversely proportional. Furthermore, as highlighted in the previous subsection, the EE increases with longer sonication time and higher amounts of FL and PVA, which correspondingly leads to an increase in LC.

### 3.2. Optimization of FL-Loaded PLGA NPs Preparation

After validating the obtained data through statistical analysis, the NPs formulation was optimized by identifying the optimal experimental conditions. The range limits for both independent and dependent variables were defined within the experimental range to ensure reliability and validity, as predicting values outside of this range could lead to inaccurate results due to the lack of experimental data. Thus, the targeted range for NP size was set between 137 nm and 190 nm, with the goal of achieving the smallest possible NPs. For zeta potential, the aim was also to minimize this parameter, with a target range between −19.2 mV and −11.3 mV. The EE was set to remain within 52% to 75%, and the LC was to be maximized up to 8%. Based on these criteria, optimal experimental values for the independent variables were as follows: 28.9 mg of PLGA, 2.0 mg of FL, 5.0% (*w*/*v*) of PVA, and a sonication cycle of 20.0 s. To ensure the consistency and reliability of the results, the optimal formulation was prepared in triplicate. The obtained results and the predicted values are presented in Table 2.

Analysis of Table 2 revealed that the experimental values fall within the range predicted by the software, thus validating the model. Additionally, the optimized NPs exhibited characteristics that are appropriate for brain tumor delivery, with sizes below 200 nm and a negative zeta potential value. According to other authors, NPs larger than 200 nm are rapidly cleared by the phagocytic system, while those smaller than 200 nm are better suited for tumor targeting [53]. Although the relevance of the EPR effect in human tumors remains debated, this size range is also favorable for passive targeting mechanisms, due to the abnormal vasculature typically observed in GBM [54,55,56]. The obtained PdI value (≤0.2) indicates that the NPs are monodisperse, which is suitable for drug delivery.

Furthermore, the toxicity and stability of NPs are influenced by their zeta potential. Negatively charged NPs are reported to be less toxic than positively charged ones and exhibit greater stability than neutral-charged NPs [57,58]. In addition, negatively charged NPs tend to exhibit lower nonspecific interactions with plasma proteins and cells, which helps in improving their systemic stability and biocompatibility [55,56]. Although the EPR effect is currently being debated, reports have indicated that negatively charged NPs can prolong circulation time and enhance tumor accumulation via this effect [59].

Similar criteria have been adopted by several authors in the design of PLGA-based nanocarriers for GBM therapy, showing promising results in terms of stability, in vivo brain accumulation, and in vivo therapeutic efficacy [60,61].

Additionally, besides electrostatic stabilization, PVA also confers stability to the NPs through steric interactions, creating a surface layer that prevents agglomeration [62]. Indeed, the NPs demonstrated sustained colloidal stability. The colloidal stability of the NPs under storage conditions (aqueous suspension at 4 °C) was monitored over 10 weeks. DLS measurements revealed no significant changes in size and PdI values, confirming their long-term stability as their physicochemical properties remained unchanged over time (Table S7 in the Supplementary File). A slight variation in zeta potential was observed, becoming less negative over time. However, this change is likely related to the measurement process itself, particularly the application of an electric field during analysis and is not indicative of NP degradation or aggregation.

Furthermore, the EE was also adequate, being higher than 50%. The LC values are consistent with others reported in the literature, which notes that LC values for PLGA NPs are typically low [63].

### 3.3. Effect of FA-Conjugation on NPs’ Properties

To enhance the specificity of FL-loaded NPs for brain tumors, their surface was functionalized with FA, which has a high affinity for folate receptors overexpressed on the BBB’s endothelial cells and GBM cells [23]. FA conjugation was accomplished using a carbodiimide-mediated reaction with EDC, which activates carboxyl groups on the NP’s surface. This activation produces an unstable O-acylisourea intermediate that reacts with the amine groups on FA, forming a stable amide bond and effectively conjugating FA to the FL-loaded NPs.

The physicochemical properties of the FL-loaded NPs were thoroughly assessed after surface conjugation with FA, confirming their suitability for targeting brain tumors. The FA-conjugated FL-loaded PLGA NPs presented an average diameter of 167 ± 8 nm, a PdI of 0.23 ± 0.07, and a zeta potential of −22.2 ± 0.3 mV. Interestingly, among all properties, PdI was the only one that did not significantly change with FA-conjugation (*p* > 0.05). The size increase following FA-conjugation of about 23% can be justified by the fact that new layers are present on the surface of the NPs [64]. The zeta potential also became more negative due to the negative charge of FA caused by the ionization of its hydroxyl groups.

Furthermore, it was verified that after functionalization of the NPs with FA, EE and LC decreased to 44.4 ± 3.8% and 3.1 ± 0.3%, respectively. This reduction is likely due to the loss of FL molecules from the NP surface during the agitation and centrifugation steps of the conjugation procedure.

**Figure 1:**
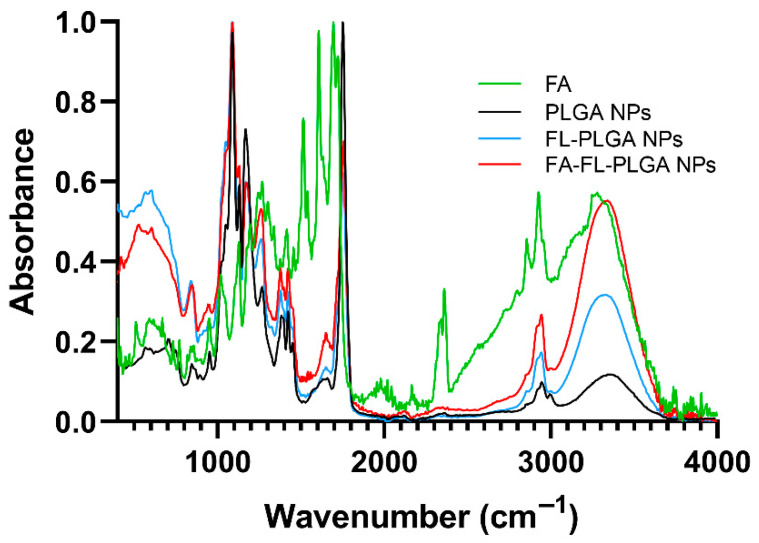
FTIR spectra of FA-conjugated and non-conjugated FL-loaded PLGA NPs, PLGA, and FA stock solution.

The observed variations in the NPs’ physicochemical properties suggest that FA was efficiently conjugated to the NPs’ surface. Similar changes in NPs size and zeta potential after surface modification with targeting ligands such as FA have also been reported by other authors as indirect evidence of successful conjugation [65]. However, to further confirm this, FTIR analysis was employed, and the attained results are presented in Figure 1.

Figure 1 shows that both the developed FA-conjugated and non-conjugated FL-loaded NPs displayed the characteristic peaks of PLGA, such as 3450–3500 cm^−1^ corresponding to the OH end group, 2885–3010 cm^−1^ for the C–H stretches, 1762 cm^−1^ corresponding to the C=O stretch, 1186–1089 cm^−1^ for the C–O stretch, and 1450–850 cm^−1^ corresponding to C–H bends [66].

Also, the FTIR spectrum of the FA-conjugated FL-loaded NPs shows characteristic peaks of FA, although at a lower intensity compared to the free FA spectrum, indicating the presence of FA on the NPs’ surface. These characteristic bands, observed between 1500 and 1700 cm^−1^, correspond to phenyl and pterin ring vibrations (1485–1519 cm^−1^), NH bending (1604 cm^−1^), C=C aromatic stretching (1619 cm^−1^), C=N stretching (1639 cm^−1^), C=O amide stretching (1650 cm^−1^), and C=O carboxylic stretching (1693 cm^−1^) [67]. Additionally, a broad peak is observed in the region of 3100–3500 cm^−1^, which corresponds to the OH groups of the carboxylic moiety of the glutamic acid in FA and the NH group stretching of the pterin ring. This peak overlaps with a characteristic PLGA peak at 3450–3500 cm^−1^ from the OH end group. In the FA-conjugated NPs, the increased intensity in this region suggests a combination of both FA and PLGA contributions, further supporting successful conjugation. Moreover, slight shifts in the characteristic FA bands were observed in the FTIR spectra of the conjugated NPs when compared to the free FA. These spectral changes are consistent with a covalent conjugation process and not merely a physical adsorption of FA onto the NP surface.

It was also verified that the spectra of PLGA and FL-loaded PLGA NPs are very similar, suggesting that the drug is primarily located within the matrix of the NPs rather than on their surface. However, several bands that are common to PLGA appear with increased intensity, likely due to the overlap with characteristic peaks of FL. These peaks include C=C stretching at 1616 cm^−1^, C–F stretching at 1250 cm^−1^, ring deformation at 650 cm^−1^, CC in-plane bending at 520 cm^−1^, and CCC out-of-plane bending at 475 cm^−1^ [68]. This effect is less pronounced in the FA-functionalized NPs, suggesting that some FL molecules adsorbed on the surface were removed during the conjugation process, as mentioned above. The spectra of free FL can be found in the Supplementary Material (Figure S5).

Moreover, despite the variations in the NPs’ physicochemical properties, the incorporation of FA did not adversely impact the colloidal stability of the NPs nor their morphology. In fact, in vitro stability studies revealed that no significant changes occurred in size, PdI, and zeta potential values, confirming that the NPs maintained their integrity for up to 10 weeks under storage conditions (Table S8 in the Supplementary Material).

**Figure 2:**
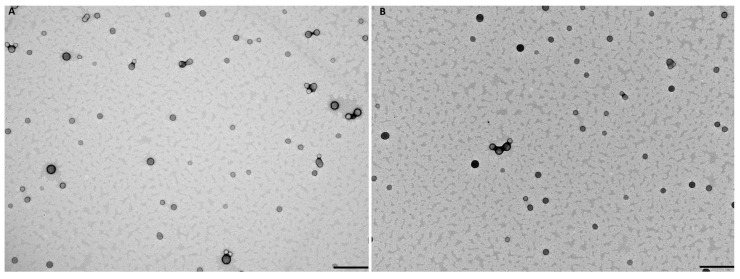
TEM image of (**A**) FL-loaded PLGA NPs and (**B**) FA-FL-loaded PLGA NPs. Scale bar: 500 nm.

Additionally, TEM analysis in Figure 2 demonstrates that both non-conjugated and FA-conjugated FL-loaded PLGA NPs present a spherical and uniform shape. According to other works, the biggest advantage of producing spherical NPs is their higher uptake, when compared to other shapes, like rod or elliptical [69]. However, this depends on the size of NPs and the type of cells used. Additionally, the observed sizes were slightly smaller than those obtained by DLS. This discrepancy occurs due to the fact that DLS measures the hydrodynamic size of NPs. The hydrodynamic size is influenced by factors such as polydispersity and hydration layers around the NPs, leading frequently to an overestimation of the NPs’ size in DLS measurements [70].

### 3.4. FL Release Kinetics from NPs

**Figure 3:**
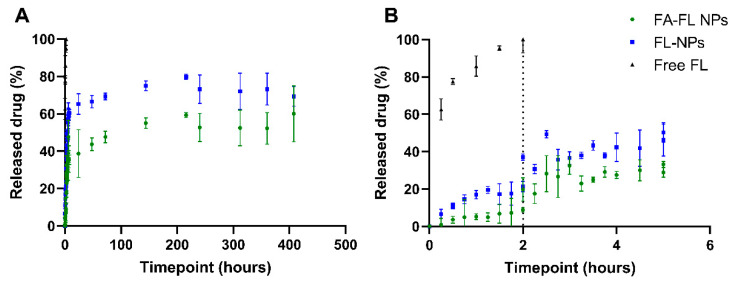
Release profile of FA-conjugated and non-conjugated FL-loaded PLGA NPs in simulated digestion conditions. Free FL was used as a control. The samples were incubated for 2 h in FaSSGF (pH 1.2), followed by 3 h in FaSSIF (pH 6.5), and then incubated in PBS (0.01 M, pH 7.4) until the end of the experiment. All data are presented as mean and standard deviation (*n* = 3). (**A**) Complete cumulative release curve over the full experimental period (408 h, 17 days). (**B**) Magnified view of the first 5 h of the experiment (simulating in vivo digestion). The vertical dashed line indicates the medium change from gastric to intestinal simulated fluids.

The in vitro release of FL was evaluated under conditions that simulate the gastrointestinal environment following oral administration, using biorelevant media. This administration route was selected considering that FL is already clinically used via the oral route, making it a realistic and translatable approach for future therapeutic applications. To mimic gastric and intestinal fluids, FaSSGF and FaSSIF were employed, respectively. This approach provides a more accurate estimation of gastrointestinal dissolution, which is often overestimated when using simple buffer systems. Besides pH, other physiological parameters such as bile and pancreatic secretions, surface tension, osmolality, and fluid volume in the gastrointestinal tract significantly affect drug solubility and dissolution behavior [57]. These components are essential in promoting the dissolution of poorly soluble compounds. The chosen media include naturally occurring micelles formed by bile salts and phospholipids (lecithin), reflecting fasted-state physiological conditions [58]. After this initial phase, the NPs were transferred to PBS to simulate systemic conditions, accounting for physiological pH, osmolarity, and ionic composition [59]. The resulting release profiles of FL-PLGA NPs, both FA-conjugated and non-conjugated, are shown in Figure 3.

As illustrated in Figure 3, after 2 h in gastric medium, FL release was 8.9 ± 1.2% for FA-conjugated NPs and 21 ± 3% for non-conjugated NPs, whereas free FL exhibited complete release (100%) within 2 h. After 3 h in a simulated intestinal medium, FL release increased to 33 ± 1% and 37 ± 2% for FA-conjugated and non-conjugated NPs, respectively. These results highlight the advantage of NP encapsulation in prolonging FL release compared to the free drug.

However, a significant fraction of the drug is still lost before reaching systemic circulation in both NP formulations. This burst release is attributed to the desorption of FL molecules from the NP surface. As expected, the surface conjugation with FA altered the release profile, as the functionalization process may reduce the amount of surface-adsorbed FL molecules, leading to a less pronounced burst effect. This burst release aligns with previous reports on PLGA-based drug delivery systems (DDS), which emphasize the challenge of minimizing premature drug loss in oral administration [71]. However, the poor oral absorption of free FL due to extensive first-pass metabolism reduces its bioavailability to below 90% [72]. The drug encapsulation in PLGA NPs remains a promising strategy to enhance drug delivery and prolong systemic circulation.

Following the initial burst release, both formulations exhibited a slower release pattern. However, FA-conjugated FL-PLGA NPs demonstrated a more prolonged and controlled release compared to non-conjugated FL-PLGA NPs. Particularly, while non-conjugated PLGA NPs achieved a FL release of about 69% within 17 days, FA-conjugated NPs released 60% of the drug in the same period. This difference can be attributed to the presence of FA on the NPs’ surface, which likely hinders water permeation, reducing PLGA hydrolysis and consequently limiting FL diffusion [24]. To better understand this observation, the colloidal stability of both formulations was monitored using DLS measurements. The results revealed that while non-conjugated NPs exhibited a gradual decrease in size over time, FA-conjugated NPs maintained a relatively stable hydrodynamic diameter (Tables S9 and S10 in the Supplementary Material), further supporting the role of FA in prolonging NPs’ hydrolysis and FL release.

This extended release period of 17 days was selected because it is expected that, although NPs are rapidly cleared from the systemic circulation, they can accumulate and remain in tumor tissue for prolonged periods. For instance, a study by Kalman et al. demonstrated that FITC-glycol chitosan NPs accumulated in murine melanoma tumors up to 14 days after intravenous injection [73]. Particularly, tumoral accumulation increased gradually over 11 days, reaching 25.9% of the injected dose per gram of tissue, and although no further increase was observed after this point, the NPs remained present in the tumor at day 14, while decreasing in other organs.

To further evaluate the release kinetics, the experimental data were fitted to different mathematical models, and the kinetic parameters and model fitting results are presented in Table S11 in the Supplementary Material. The Korsmeyer–Peppas model provided the best fit for both FA-conjugated and non-conjugated NPs, as indicated by the highest correlation coefficients (R^2^). The obtained *n* values were 0.768 for FA-conjugated NPs and 0.759 for non-conjugated NPs. The obtained exponent *n* provides information about the drug release mechanism. Values between 0.43 and 0.85, such as the ones obtained in this experiment for both formulations, are indicative that FL release follows a predominantly anomalous (non-Fickian) diffusion mechanism. These results indicate that the FL release is controlled by a combination of Fickian diffusion through the polymer matrix and a time-dependent process related to the erosion of the matrix [74]. This hybrid mechanism is typical for PLGA-based systems and is advantageous for achieving controlled and sustained release profiles.

Additionally, as expected, the kinetic constant (*K*) was higher for FL-NPs compared to FA-FL NPs, indicating that the presence of FA slows down FL release as mentioned above.

These findings suggest that FA-conjugated NPs offer a controlled and prolonged release of FL under simulated physiological conditions, making them a promising nanosystem for FL delivery. Nonetheless, in addition to their sustained release behavior, further improvements could be achieved by tailoring the system to respond to specific tumor microenvironment conditions. Tumor tissues typically exhibit a slightly acidic microenvironment (pH ~6.8), which can influence the behavior of polymeric NPs. Therefore, the development of pH-responsive PLGA-based NPs may be a promising strategy, using approaches such as the incorporation of acid-labile bonds, pH-sensitive surfactants, charge-conversion moieties, or pH-triggered linkers to enhance selective drug release in tumor sites [75].

### 3.5. Quantitative Analysis of NP’s Cell Uptake

**Figure 4:**
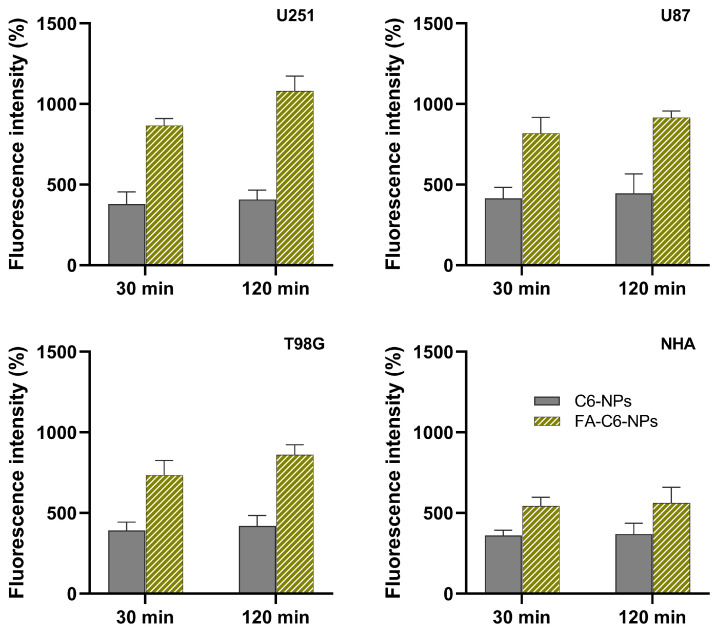
Analysis of cellular uptake by measuring the fluorescence intensity of C6-labeled NPs after incubation periods of 30 and 120 min across different cell lines. The control bars represent the autofluorescence of untreated cells. The results are expressed as a percentage relative to the autofluorescence of untreated control cells (set as 100%) and presented as mean and standard deviation (*n* = 3).

To determine whether FA-conjugation enhances cell uptake, the internalization of FA-functionalized and non-modified NPs was assessed in healthy astrocytes (NHA) and human GBM cells (U251, U87, and T98G). To quantify their internalization, the NPs were marked with a fluorescent molecule (C6). To evaluate the impact of incubation time on the uptake of NPs, the cells were incubated with the NPs for different experiment durations (30 min and 120 min). The attained results are shown in Figure 4.

The results showed that FA conjugation significantly increased NP internalization across all studied cell lines compared to non-conjugated NPs (*p* < 0.05). This enhanced uptake was particularly more expressive in GBM tumor cells compared to healthy astrocytes, likely due to the overexpression of folate receptors on rapidly proliferating tumor cells. Indeed, the expression of folate receptors in GBM cells is much higher than in healthy astrocytes, reflecting their increased folate demand to support rapid cell proliferation [76].

It was verified that after 2 h of incubation, FA conjugation led to a 34% increase in NPs’ uptake in healthy astrocytes. Specifically, the fluorescence intensity relative to untreated control cells increased from 369% for non-conjugated NPs to 560% for FA-conjugated NPs. In contrast, in tumor cells, the effect of FA conjugation on the uptake increase was even more pronounced. Compared to non-conjugated NPs, FA-conjugated NPs resulted in uptake increases of 62% in U251 cells (from 408% to 1080%), 51% in U87 cells (from 445% to 916%), and 51% in T98G cells (from 419% to 860%). Additionally, longer incubation times led to higher uptake rates.

**Figure 5:**
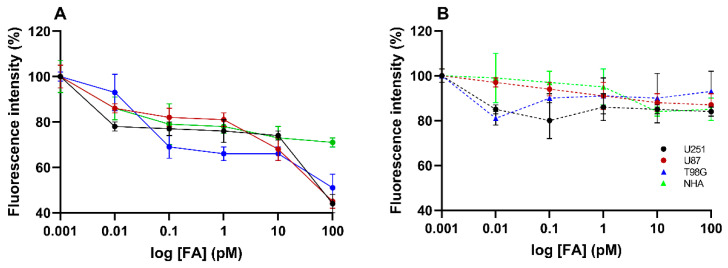
Evaluation of the effect of folate receptor saturation on the uptake of C6-labeled NPs across different cell lines. The cells were pre-treated with increasing concentrations of free FA for 1 h, followed by a 2-h incubation with FA-conjugated or non-conjugated C6-labeled NPs. (**A**) Results for FA-conjugated NPs are represented by solid lines, and (**B**) results for non-conjugated NPs are shown with dashed lines. All results are presented as mean and standard deviation (*n* = 3).

To further assess the mechanism involved in NPs’ uptake, a folate receptor saturation assay was conducted. For that, the cells received a pre-treatment of free FA at increasing concentrations for 1 h before incubation with NPs. The aim was to block the folate receptor and assess its effect on NPs’ uptake. The attained results are depicted in Figure 5.

The results demonstrated that increasing concentrations of free FA led to a proportional decrease in the internalization of FA-conjugated NPs (*p* < 0.05), while non-conjugated NPs showed no significant change (*p* > 0.05). For example, in U251 cells, at the highest FA concentration (100 pM), the uptake of FA-conjugated NPs decreased to 44%, compared to a decrease to 84% for non-conjugated NPs. Similarly, in T98G cells, FA-conjugated NP uptake decreased to 51%, while non-conjugated NPs showed only a slight decrease to 93%.

These findings corroborate the above-mentioned results, indicating that FA-conjugated NPs exhibit greater selectivity for tumor cells over healthy astrocytes. In fact, folate receptor saturation had a more pronounced inhibitory effect on NPs’ internalization in tumor cells compared to healthy astrocytes. Specifically, after treatment with free FA at a concentration of 100 pM, the uptake of FA-conjugated NPs in astrocytes only decreased to 71%.

Overall, these results demonstrate that FA-conjugated NPs exhibit significantly enhanced uptake in GBM cells compared to healthy astrocytes, confirming the ability of this delivery system to selectively target tumor cells. The stronger inhibitory effect observed in GBM cells following folate receptor saturation, when compared to NHA, further demonstrates the role of receptor-mediated endocytosis in the uptake mechanism. These findings support that this conjugation not only enhances NP accumulation in tumor cells but also increases selectivity for these cells, minimizing off-target effects on healthy brain tissue and providing a promising strategy for targeted GBM therapy.

Although the EPR effect has historically justified the development of NPs for cancer therapy, recent evidence indicates that this mechanism may play a limited role, even in preclinical models. For instance, a study by Sindhwani et al. (2020) demonstrated that large inter-endothelial gaps, previously considered the main route for NPs extravasation, are far less frequent than previously believed and insufficient to account for the observed levels of tumor accumulation [77]. In contrast, active trans-endothelial transport mechanisms appear to be responsible for up to 97% NPs’ uptake into tumor tissue. These findings demonstrate the relevance of using active targeting strategies to improve NP delivery. In this context, the system developed in this work was designed to combine passive and active targeting, relying on folate receptor-mediated endocytosis to enhance tumor selectivity and overcome the limitations associated with the heterogeneity and unpredictability of the EPR effect.

### 3.6. Cytotoxicity Evaluation of FL-Loaded NPs

**Figure 6:**
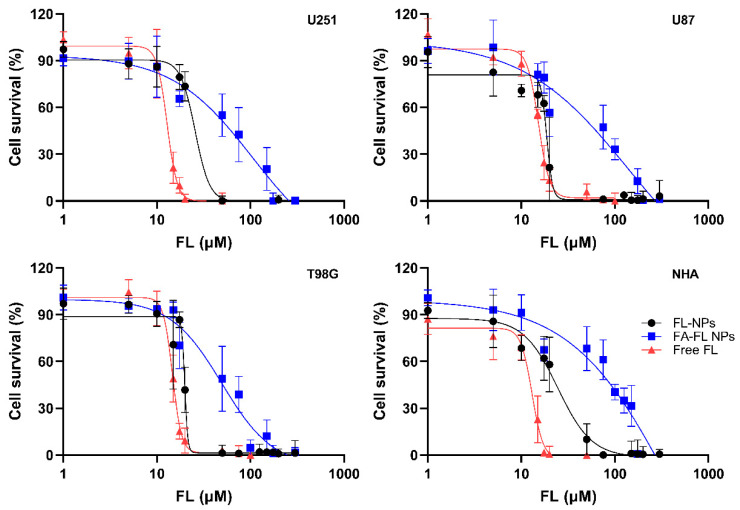
Dose–response curves for different GBM cell lines (U251, U87, T98G) and healthy astrocytes (NHA) using the SRB assay after 72-h treatment with free FL, FA-FL-loaded, or FL-loaded NPs. The results are presented as mean and standard deviation (*n* = 3).

The antiproliferative activity of the developed FL-loaded NPs in comparison with the free drug was evaluated across different GBM cell lines (U251, U87, and T98G) and healthy astrocytes, which served as controls. To determine whether the FA conjugation offers any advantages, both FA-conjugated and non-conjugated NPs were tested. The dose–response curves generated from the obtained results are presented in Figure 6.

Figure 6 depicts that, regardless of whether the drug is in its free form or encapsulated within either conjugated or non-conjugated NPs, its inhibitory effect is dose-dependent, with greater inhibition observed at higher drug concentrations. From these dose–response curves, the IC50 values were calculated, which represent the concentration of the drug required to inhibit cell proliferation by 50%. The determined IC50 values are listed in Table 3 below.

The IC50 values obtained for the GBM cells are within the range reported in the literature for free FL (12–30 µM) [17,78]. Interestingly, contrary to other studies that found that 25–30 μM FL has no significant effect on the viability of healthy astrocytes [79], these results show that FL exhibits as similar antiproliferative activity in NHA as it does in cancer cells (*p* < 0.05 in comparison with GBM cell lines).

Additionally, the IC50 values suggest that the free drug is more effective at reducing cell viability in all cell lines compared to the encapsulated drug in both NP formulations. However, these results can be justified by the slow release of the drug from the NPs, as previously mentioned. Over the 72-h duration of this experiment, only a fraction of the total encapsulated FL is expected to be released, meaning that the entire drug was not yet available to exert its full therapeutic effect. Thus, this does not necessarily indicate a lower efficacy of the NPs but indicates a controlled release behavior, which is expected to be advantageous in vivo by ensuring prolonged drug availability and potentially reducing systemic toxicity.

Moreover, the results allow us to draw conclusions about the observed differences between conjugated and non-conjugated NPs. For all cell lines tested, the efficacy of the FA-conjugated NPs appears to be lower than that of the non-conjugated NPs, as indicated by the higher IC50 values (*p* < 0.05). This observation could also be attributed to the slower release rate of the drug from the conjugated NPs, which may be partially hindered by the FA on the NP surface, as previously discussed.

Furthermore, FA conjugation also proved beneficial in enhancing the specificity of the NPs toward tumor cells, as suggested by the IC50 values obtained. While the IC50 values are similar across all cell lines for non-conjugated NPs, indicating comparable internalization across different cell types, this is not the case for the FA-conjugated NPs. For the FA-conjugated NPs, the IC50 for NHA cells is significantly higher (*p* < 0.05), suggesting that due to the lower expression of the folate receptor on these healthy cells, the NPs are less internalized than in GBM cells, thereby potentially sparing healthy cells from the cytotoxic effects. Additionally, it is important to consider that this advantage could be even more pronounced under in vivo conditions, where the tumor microenvironment and systemic factors could further enhance the targeting specificity and reduce off-target effects due to the EPR effect and receptor-mediated endocytosis in the tumor tissue.

Moreover, it was demonstrated that the materials used for the production of the NPs are biocompatible, as both the non-conjugated and conjugated unloaded NPs (controls) were tested at concentrations ranging from 50 to 300 µg/mL across all four cell lines and did not exhibit any deleterious effects on the cells. This indicates that the NP formulation is a safe vehicle for potential GBM treatment (Figure S6 in the Supplementary Information File).

Relevant performance metrics commonly used to evaluate NPs as DDS, such as drug LC and drug release rate, are important parameters to better understand the therapeutic outcomes observed in this study [80]. For instance, while higher release rates are often associated with enhanced cytotoxic effects in vitro, this relationship is not universal. In fact, sustained release profiles, such as the one exhibited by these FL-PLGA NPs, are often preferable in conventional chemotherapy approaches, if drug concentrations remain above the therapeutic threshold in the tumor microenvironment. Thus, despite presenting a prolonged release and exhibiting slightly higher IC50 values compared to free FL (ranging between 18 and 78 µM versus 12–15 µM), they still fall within a therapeutically relevant range and demonstrated enhanced selectivity toward tumor cells due to folate receptor targeting. These results suggest that the slower release profile does not necessarily compromise in vitro efficacy and may instead contribute to the sustained intracellular availability of FL, which is expected to be advantageous in vivo.

It is also important to consider that PLGA-based NPs are known to have lower LC compared to other DDS, which may limit the amount of drug delivered per dose. However, this drawback is balanced by other advantages, including excellent biocompatibility, biodegradability, and the fact that PLGA is FDA-approved for clinical use.

However, LC values are highly dependent on the nature of both the carrier and the drug, making direct comparisons only meaningful when the same drug is being evaluated. To the best of our knowledge, no previous studies have reported PLGA-based NPs loaded with FL, regardless of the therapeutic application. Therefore, here a comparison is made with a recent study by Shoaib et al. (2025), where polymeric dextran-based NPs loaded with FL were developed [81]. In that system, the LC ranged from 44 to 48%, depending on the FL: polymer ratio. In U87 GBM cells, treatment with 30 µM FL-loaded dextran NPs for 5 h resulted in a reduction in cell viability to 20–50%, depending on formulation. In our system, using a longer incubation time (72 h), IC50 values of 18.1 µM and 52.7 µM were obtained for non-conjugated and FA-conjugated NPs, respectively. Thus, the results of this present work are within a comparable range, despite the much lower LC (3.1% for FA-conjugated and 3.6% for non-conjugated NPs). Regarding drug release, the dextran NPs exhibited a release of 25–40% after 96 h at pH 7.4, depending on the FL:dextran ratio. In comparison, the FL-NPs developed in this present work showed faster release rates of 47% for FA-conjugated and 69% for non-conjugated formulations within just 72 h. Despite these differences in release kinetics and LC, the anticancer activity remained comparable, demonstrating the efficiency of the system developed in the present work.

Overall, the results highlight the potential of FA-conjugated NPs to enhance the selectivity of FL delivery toward GBM cells. While the controlled release of FL from the NPs led to a slightly reduced cytotoxic effect in vitro compared to the free drug, this behavior is expected to be advantageous in vivo, where prolonged drug availability can enhance therapeutic efficacy and reduce systemic toxicity. Moreover, the higher IC50 values observed in healthy astrocytes for FA-conjugated NPs, as opposed to the comparable values across all cell lines for non-conjugated NPs, demonstrate that FA-conjugation promotes selective uptake in tumor cells. Taken together, these findings support the rationale for using FA-modified PLGA NPs as a targeted strategy to improve tumor specificity while minimizing undesired effects on healthy tissues.

### 3.7. Enhancement of TMZ Sensitivity in GBM Cells by FL-Loaded NPs

To determine if the developed FL-loaded NPs could sensitize GBM cells to the standard drug used in the treatment of this disease, the alkylating agent TMZ, a combined therapy assay with TMZ and the NPs, was performed. For these experiments, three different GBM cell lines were used, with different levels of expression of the MGMT protein, which influences their sensitivity to alkylating agents. It is well-documented that the U251 cell line has low MGMT expression, making these cells sensitive to TMZ. In contrast, the U87 cell line exhibits moderate resistance, while the T98G cell line is highly resistant to TMZ due to the overexpression of the DNA repair protein MGMT [82].

**Figure 7:**
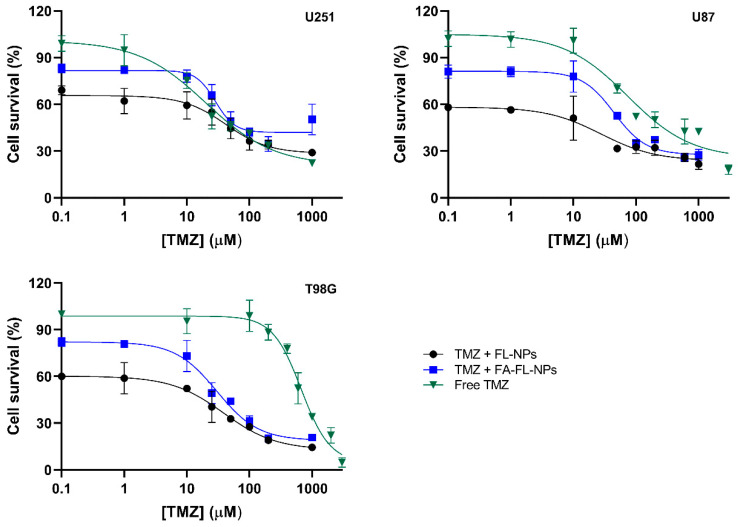
Dose–response curves for different GBM cell lines (U251, U87, T98G) using the SRB assay after 72-h treatment with free TMZ and combination therapy of free TMZ and FA-FL-loaded or free TMZ and FL-loaded NPs. The results are presented as mean and standard deviation (*n* = 3).

The cells were treated for 72 h with increasing concentrations of TMZ (ranging from 0.1 to 3000 µM) combined with a fixed concentration of FA-conjugated or non-conjugated FL-loaded NPs (15 µM for GBM cells and 25 µM for healthy astrocyte cells). The concentration of NPs used was based on the IC80 value determined in previous assays. As a control, cells were also treated with TMZ alone. The attained results are presented in Figure 7 below.

The IC50 values obtained (Table 4) are within the range reported in the literature for TMZ in its free form [83,84,85]. Statistical analysis confirmed that the differences in IC50 values among the three GBM cell lines are significant (*p* < 0.05). These differences can be attributed to their different levels of MGMT protein expression. As MGMT repairs TMZ-induced DNA lesions, cell lines with higher MGMT expression, such as T98G, exhibit increased resistance to this drug, whereas cell lines with lower expression, such as U251, are more sensitive.

As expected, it was observed that for the U251 TMZ-sensitive cells, the combination with FL-nanoformulations did not alter the IC50 values (*p* > 0.05). However, for the TMZ-resistant cell lines (U87 and T98G), treatment with TMZ in combination with both of the FL-loaded NP formulations (FA-conjugated or non-conjugated) led to a statistically significant reduction in IC50 values (*p* < 0.05) compared to treatment with TMZ alone. Also, for U87 and T98G cells, the FA-conjugated NPs appeared slightly less effective than the non-conjugated NPs. This observation may be explained by the slower release profile of the FA-modified formulation, as previously discussed.

These findings further support the potential of the developed FL-loaded NPs to act as chemosensitizers in GBM therapy. This is because the action of FL is independent of the DNA repair mechanisms mediated by MGMT. Therefore, the developed NPs have the potential to be a promising strategy to enhance the effectiveness of GBM treatment by overcoming the limitations of MGMT-mediated drug resistance. Additionally, since MGMT overexpression is also observed in other cancers with aberrant promoter methylation, these NPs may hold potential for application in the treatment of other difficult-to-treat tumors such as lung, ovarian, and breast cancer [86,87,88]. Furthermore, as the developed NPs can be adapted to carry different therapeutic agents, this system could be valuable in other combination regimens beyond GBM.

## 4. Conclusions

Current treatments for GBM are not curative, with poor prognosis and high mortality rates due to their inability to overcome intrinsic resistance, such as the DNA repair MGMT protein, which contributes to tumor resistance. This creates an urgent need for new and more effective therapies. Nanotechnology offers a promising solution, with the potential for enhanced specificity and precision in targeting tumor cells, improved drug solubility, reduced risk of drug resistance, and minimized systemic toxicity. In this work, an optimized PLGA-based formulation for FL encapsulation was successfully developed and was further functionalized with FA to increase its targeting ability and specificity for brain tumor cells. The in vitro studies demonstrated that FA functionalization significantly enhanced the NPs’ accumulation in the target GBM cells, while also improving selectivity by reducing uptake in healthy astrocytes. This specificity may be attributed to a folate receptor-mediated endocytosis mechanism, since this receptor is overexpressed in tumor cells, allowing for a more targeted delivery approach. Furthermore, the formulated NPs not only preserved the antiproliferative effect of FL but were also able to sensitize tumor cells to the gold standard (TMZ) in GBM therapy. Overall, the findings of this research suggest that FA-conjugated FL-loaded NPs could be a powerful strategy for GBM treatment, offering targeted drug delivery and enhanced therapeutic efficacy. By improving drug accumulation in tumor cells while minimizing effects on healthy cells, these NPs hold great potential as a novel and effective option in the fight against this devastating disease.

## Figures and Tables

**Table 1:** Independent levels and their experimental range applied for the CCD.

Independent Variable	Units	Experimental Level				
		−α ^1^	−1	0	+1	+α ^1^
mPLGA	mg	3.8	10.0	25.0	40.0	46.2
mFL	mg	0.2	0.5	1.25	2.0	2.3
% PVA	% (*w*/*v*)	0.2	1.0	3	5	5.8
Sonication Cycles	s	1.9	5	12.5	20	23.1

^1^ 1.54671 was implemented as the α value for considering a factorial orthogonal quadratic design.

**Table 2:** Experimental values and predicted values by the software. The results are presented as mean and standard deviation (*n* = 3).

		Predicted Values	Experimental Values
**Size (nm)**	**Mean value**	119	136 ± 2
	**Range**	(66–172)	(134–138)
**PdI**	**Mean value**	0.22	0.22 ± 0.04
	**Range**	(0.02–0.45)	(0.18–0.26)
**Zeta potential (mV)**	**Mean value**	−15.9	−14.6 ± 1.4
	**Range**	(−18.0–[−13.8])	(−16.1–[−13.4])
**EE (%)**	**Mean value**	52	52.3 ± 2.4
	**Range**	(42.7–61.3)	(50.5–55.0)
**LC (%)**	**Mean value**	4.1	3.6 ± 0.2
	**Range**	(3.3–4.9)	(3.5–3.8)

**Table 3:** IC50 values derived from the dose–response curves for different GBM cell lines (U251, U87, T98G) and healthy astrocytes (NHA) using the SRB assay after 72-h treatment with free FL, FA-FL-loaded, or FL-loaded NPs. The results are presented as mean and standard deviation (*n* = 3).

	IC50 (µM)			
	**U251**	**U87**	**T98G**	**NHA**
**Free FL**	12.8 ± 2.0	15.2 ± 0.6	14.8 ± 1.8	12.1 ± 2.3
**FL-loaded PLGA NPs**	24.7 ± 3.1	18.1 ± 1.2	19.7 ± 3.0	21.5 ± 0.9
**FA-FL-loaded PLGA NPs**	52.5 ± 5.3	52.7 ± 4.5	45.0 ± 0.9	77.9 ± 6.2

**Table 4:** IC50 values derived from the dose–response curves for different GBM cell lines (U251, U87, T98G) using the SRB assay after 72-h treatment with free TMZ and combination therapy of free TMZ and FA-FL-loaded or free TMZ and FL-loaded NPs. The results are presented as mean and standard deviation (*n* = 3).

	IC50 (µM)		
	**U251**	**U87**	**T98G**
**Free TMZ**	40.5 ± 4.8	189 ± 15	699 ± 29
**Free TMZ + FL-loaded PLGA NPs**	34.8 ± 5.1	7.1 ± 6.8	11.2 ± 1.5
**Free TMZ + FA-FL-loaded PLGA NPs**	49.6 ± 7.4	53.9 ± 1.7	31.0 ± 3.1

## Data Availability

Data are unavailable due to privacy restrictions.

## Supplementary Materials

The following supporting information can be downloaded at https://www.mdpi.com/article/10.3390/pharmaceutics17091116/s1, Table S1: Experimental plan; Tables S2–S6: ANOVA results for size, PdI, zeta potential, EE, and LC; Regression Equations (S1)–(S4): Predictive models for size, zeta potential, EE, and LC; Figures S1–S4: Contour and surface plots for the effect of formulation variables; Tables S7 and S8: Colloidal stability under storage conditions; Figure S5: FTIR spectrum of free FL; Tables S9 and S10: Colloidal stability under simulated digestion conditions; Table S11: Drug release parameters; Figure S6: Cell viability results for unconjugated and FA-conjugated NPs.

## Abbreviations

The following abbreviations are used in this manuscript:

ATR-FTIR	Attenuated total reflectance Fourier-transform infrared spectroscopy
CCD	Central Composite Design
CNS	Central Nervous System
C6	Coumarin-6
DMEM	Dulbecco’s Modified Eagle Medium
DDS	Drug Delivery Systems
DLS	Dynamic Light Scattering
DMSO	Dimethyl Sulfoxide
EDC	N-(3-dimethylaminopropyl)-N′-ethylcarbodiimide hydrochloride
EE	Encapsulation efficiency
FA	Folic acid
FaSSGF	Fasted State Simulated Gastric Fluid
FaSSIF	Fasted State Simulated Intestinal Fluid
FBS	Fetal Bovine Serum
FL	Fluoxetine
GBM	Glioblastoma
LC	Loading capacity
MGMT	O6-methylguanine DNA methyltransferase
NPs	Nanoparticles
OAT	One factor at time
PBS	Phosphate-buffered saline
PdI	Polydispersity index
PLGA	Poly(lactic-co-glycolic acid)
PVA	Polyvinyl alcohol
SMPD1	Sphingomyelin phosphodiesterase 1
SRB	Sulforhodamine B
SSRI	Selective serotonin reuptake inhibitor
TCA	Trichloroacetic acid
TEM	Transmission electron microscopy
TMZ	Temozolomide
