# Supplementary material for: Targeted Fluoxetine Delivery Using Folic Acid-Modified PLGA Nanoparticles for Selective Uptake by Glioblastoma Cells
Source: Pharmaceutics. 2025 Aug 27;17(9):1116. doi: 10.3390/pharmaceutics17091116 (PMC12473577; doi:10.3390/pharmaceutics17091116)
Supplement: Supplementary file 1 [file pharmaceutics-17-01116-s001.zip › pharmaceutics-3829310-supplementary.pdf]

Supplementary Information

A. Central Composite Design

Table S1. Experimental plan, including the independent variables and respective outcomes.

Run	Independent variables				Response variables				
	PLGA (mg)	Fluoxetine (mg)	PVA (%)	Sonication time (s)	Size (nm)	PdI	Zeta potential (mV)	EE (%)	LC (%)
**1**	46.2	1.25	3.0	12.5	157.8	0.251	-13.3	56.3	1.50
**2**	10.0	0.5	5.0	20.0	168.9	0.395	-17.7	32.1	1.60
**3**	25.0	1.25	3.0	23.1	154.7	0.176	-16.3	44.1	2.20
**4**	10.0	0.5	1.0	20.0	166.9	0.194	-18.6	37.3	1.90
**5**	25.0	0.2	3.0	12.5	189.7	0.171	-19.2	8.3	0.10
**6**	40.0	2.0	5.0	5.0	268.1	0.322	-14.5	58.4	2.90
**7**	10.0	2.0	5.0	5.0	184.1	0.277	-13.6	33.6	6.70
**8**	10.0	2.0	1.0	20.0	189.6	0.312	-14.7	30.3	6.10
**9**	3.8	1.25	3.0	12.5	198.1	0.376	-17.3	17.3	5.70
**10**	10.0	2.0	5.0	20.0	136.7	0.316	-11.3	43.3	8.70
**11**	40.0	2.0	5.0	20.0	189.7	0.314	-18.5	57.3	2.90
**12**	40.0	2.0	1.0	5.0	215.2	0.346	-13.1	44.5	2.20
**13**	40.0	2.0	1.0	20.0	228.7	0.225	-13.5	41.9	2.10
**14**	40.0	0.5	5.0	20.0	138.47	0.24	-17.3	44.5	0.60
**15**	25.0	1.25	0.2	12.5	379.3	0.675	-16.6	21.1	1.10
**16**	10.0	2.0	1.0	5.0	246.2	0.424	-15.4	31.2	6.20
**17**	40.0	0.5	5.0	5.0	157.4	0.261	-15.8	27.1	0.30
**18**	40.0	0.5	1.0	20.0	204.7	0.235	-13.9	48.9	0.60
**19**	25.0	1.25	3.0	12.5	141.1	0.23	-16.2	47.9	2.40
**20**	10.0	0.5	5.0	5.0	189	0.433	-18.1	34.5	1.70
**21**	25.0	1.25	5.8	12.5	157.2	0.371	-18.9	55	2.80
**22**	25.0	1.25	3.0	12.5	142.2	0.157	-16.6	50.4	2.50
**23**	25.0	1.25	3.0	12.5	160.1	0.143	-15.6	55.4	2.80
**24**	25.0	2.3	3.0	12.5	146.8	0.179	-12.7	39.3	3.60
**25**	25.0	1.25	3.0	1.9	186.2	0.338	-17.7	43.2	2.20
**26**	10.0	0.5	1.0	5.0	173.9	0.187	-17.6	27.4	1.40
**27**	40.0	0.5	1.0	5.0	221.6	0.181	-17.0	40.9	0.50

Table S2. ANOVA analysis for the response variable size.

Source	Sum of Squares	df	Mean Square	F-value	p-value
**Model**	66886.32	24	2786.93	24.53	0.0399
A-PLGA	812.04	1	812.04	7.15	0.1160
B-Fluoxetine	922.78	1	922.78	8.12	0.1042
C-PVA	24670.87	1	24670.87	217.17	0.0046
D-Sonication	494.24	1	494.24	4.35	0.1723
AB	923.70	1	923.70	8.13	0.1041
AC	21.69	1	21.69	0.1909	0.7048
AD	57.65	1	57.65	0.5074	0.5501
BC	9.17	1	9.17	0.0807	0.8031
BD	701.85	1	701.85	6.18	0.1308
CD	597.44	1	597.44	5.26	0.1488
A^2	1090.10	1	1090.10	9.60	0.0903
B^2	502.83	1	502.83	4.43	0.1701
C^2	17419.96	1	17419.96	153.34	0.0065
D^2	616.71	1	616.71	5.43	0.1451
ABC	4775.85	1	4775.85	42.04	0.0230
ABD	143.70	1	143.70	1.26	0.3776
ACD	507.26	1	507.26	4.47	0.1689
BCD	285.86	1	285.86	2.52	0.2536
A^2B	2883.22	1	2883.22	25.38	0.0372
A^2C	13572.23	1	13572.23	119.47	0.0083
A^2D	36.58	1	36.58	0.322	0.6276
AB^2	1964.97	1	1964.97	17.30	0.0532
ABCD	785.54	1	785.54	6.91	0.1193
A^2B^2	3042.92	1	3042.92	26.79	0.0354
**Pure Error**	227.2	2	113.60		
**Cor Total**	67113.52	26			

Table S3. ANOVA analysis for the response variable PDI.

Source	Sum of Squares	df	Mean Square	F-value	p-value
**Model**	0.3414	24	0.0142	6.52	0.1414
A-PLGA	0.0078	1	0.0078	3.58	0.1990
B-Fluoxetine	0	1	0	0.0147	0.9147
C-PVA	0.0462	1	0.0462	21.17	0.0441
D-Sonication	0.0131	1	0.0131	6.01	0.1337
AB	0.0018	1	0.0018	0.8277	0.459
AC	0.0015	1	0.0015	0.6792	0.4965
AD	4.00E-06	1	4.00E-06	0.0018	0.9697
BC	0.0233	1	0.0233	10.66	0.0824
BD	0.0026	1	0.0026	1.19	0.3889
CD	0.0013	1	0.0013	0.5939	0.5215
A^2	0.0225	1	0.0225	10.3	0.0849
B^2	3.33E-06	1	3.33E-06	0.0015	0.9724
C^2	0.1439	1	0.1439	65.96	0.0148
D^2	0.0077	1	0.0077	3.55	0.2003
ABC	0.0203	1	0.0203	9.3	0.0928
ABD	0.0009	1	0.0009	0.4124	0.5865
ACD	0.0003	1	0.0003	0.1324	0.7508
BCD	0.0092	1	0.0092	4.22	0.1762
A^2B	0.0017	1	0.0017	0.762	0.4747
A^2C	0.0591	1	0.0591	27.06	0.035
A^2D	0.0064	1	0.0064	2.94	0.2286
AB^2	0.0011	1	0.0011	0.4921	0.5556
ABCD	4.00E-06	1	4.00E-06	0.0018	0.9697
A^2B^2	0.0308	1	0.0308	14.13	0.0640
**Pure Error**	0.0044	2	0.0022		
**Cor Total**	0.3458	26			

Table S4. ANOVA analysis for the response variable zeta potential.

Source	Sum of Squares	df	Mean Square	F-value	p-value
**Model**	1896.38	4	474.09	4.5	0.0083
A-PLGA	1108.82	1	1108.82	10.53	0.0037
B-Fluoxetine	419.70	1	419.70	3.99	0.0584
C-PVA	290.74	1	290.74	2.76	0.1108
D-Sonication	77.11	1	77.11	0.7322	0.4014
**Residual**	2316.85	22	105.31		
Lack of Fit	2287.85	20	114.39	7.89	0.1184
Pure Error	29	2	14.5		
**Cor Total**	4213.22	26			

Table S5. ANOVA analysis for the response variable EE.

Source	Sum of Squares	df	Mean Square	F-value	p-value
**Model**	117.30	22	5.33	9.99	0.0187
A-PLGA	8.24	1	8.24	15.44	0.0171
B-Fluoxetine	21.32	1	21.32	39.94	0.0032
C-PVA	2.65	1	2.65	4.96	0.090
D-Sonication	0.9385	1	0.9385	1.76	0.2555
AB	9.97	1	9.97	18.68	0.0124
AC	12.55	1	12.55	23.51	0.0083
AD	1.59	1	1.59	2.99	0.1591
BC	0.0163	1	0.0163	0.0305	0.8699
BD	0.3875	1	0.3875	0.7259	0.4422
CD	1.79	1	1.79	3.35	0.1411
A^2	5.57	1	5.57	10.44	0.0319
B^2	2.01	1	2.01	3.77	0.1243
C^2	1.52	1	1.52	2.85	0.1668
D^2	0.02	1	0.02	0.0375	0.8559
ABC	5.23	1	5.23	9.8	0.0352
ABD	5.65	1	5.65	10.59	0.0313
ACD	7.80	1	7.8	14.61	0.0187
BCD	0.0946	1	0.0946	0.1771	0.6955
A^2B	3.01	1	3.01	5.63	0.0765
A^2C	1.22	1	1.22	2.28	0.2058
A^2D	0.8364	1	0.8364	1.57	0.2789
AB^2	4.83	1	4.83	9.05	0.0396
**Residual**	2.14	4	0.5338		
Lack of Fit	1.62	2	0.812	3.18	0.2394
Pure Error	0.5113	2	0.2556		
**Cor Total**	119.43	26			

Table S6. ANOVA analysis for the response variable LC.

Source	Sum of Squares	df	Mean Square	F-value	p-value
**Model**	118.35	14	8.45	29.91	< 0.0001
A-PLGA	39.29	1	39.29	138.99	< 0.0001
B-Fluoxetine	58.65	1	58.65	207.48	< 0.0001
C-PVA	2.33	1	2.33	8.25	0.0140
D-Sonication	0.2637	1	0.2637	0.9329	0.3532
AB	10.66	1	10.66	37.71	< 0.0001
AC	0.2352	1	0.2352	0.8322	0.3796
AD	0.245	1	0.245	0.8668	0.3702
BC	1.38	1	1.38	4.88	0.0473
BD	0.0462	1	0.0462	0.1635	0.6930
CD	0.1806	1	0.1806	0.639	0.4396
A^2	4.94	1	4.94	17.48	0.0013
B^2	0.1585	1	0.1585	0.5609	0.4683
C^2	0.0955	1	0.0955	0.338	0.5718
D^2	0.0099	1	0.0099	0.0349	0.8550
**Residual**	3.39	12	0.2827		
Lack of Fit	3.32	10	0.3317	8.89	0.1052
Pure Error	0.0746	2	0.0373		
**Cor Total**	121.74	26			

Equation S1:

$$Size = +147.78 - 14.2482 \times A - 15.1887 \times B - 78.5348 \times C \times -11.1157 \times D + 7.59812 \times AB - 1.16438 \times AC + 1.89812 \times AD + 0.756875 \times BC - 6.62313 \times BD - 6.11063 \times CD + 15.07 \times A^2 + 10.235 \times B^2 + 60.2425 \times C^2 + 11.335 \times D^2 + 17.2769 \times ABC + 2.99688 \times ABD - 5.63062 \times ACD - 4.22687 \times BCD + 30.0168 \times A^2B + 65.1254 \times A^2C - 3.38116 \times A^2D + 24.7801 \times AB^2 - 7.00688 \times ABCD - 52.2106 \times A^2B^2$$

Legend: A - PLGA mass; B- Fluoxetine mass; C- percentage of PVA; D - Sonication time

Equation S2:

$$Zeta\ Potential = -16.557 + 1.43543 \times A + 2.3087 \times B + -0.813173 \times C + 0.484368 \times D - 0.789375 \times AB - 0.885625 \times AC - 0.315625 \times AD + 0.031875 \times BC - 0.155625 \times BD - 0.334375 \times CD + 0.794855 \times A^2 + 0.477355 \times B^2 - 0.415145 \times C^2 - 0.0476453 \times D^2 - 0.571875 \times ABC - 0.594375 \times ABD - 0.698125 \times ACD + 0.076875 \times BCD - 0.969329 \times A^2B + 0.616298 \times A^2C - 0.511243 \times A^2D - 1.22855 \times AB^2$$

Legend: A - PLGA mass; B- Fluoxetine mass; C- percentage of PVA; D - Sonication time

Equation S3:

$$EE = 39.6707 + 7.44587 \times A + 4.58095 \times B + 3.81276 \times C + 1.96355 \times D$$

Legend: A - PLGA mass; B- Fluoxetine mass; C- percentage of PVA; D - Sonication time

Equation S4:

$$LC = 2.30465 - 1.40157 \times A + 1.71244 \times B + 0.341501 \times C + 0.114828 \times D - 0.81625 \times AB - 0.12125 \times AC - 0.12375 \times AD + 0.29375 \times BC + 0.05375 \times BD + 0.10625 \times CD + 0.74843 \times A^2 - 0.13407 \times B^2 - 0.10407 \times C^2 + 0.0334302 \times D^2$$

Legend: A - PLGA mass; B- Fluoxetine mass; C- percentage of PVA; D - Sonication time

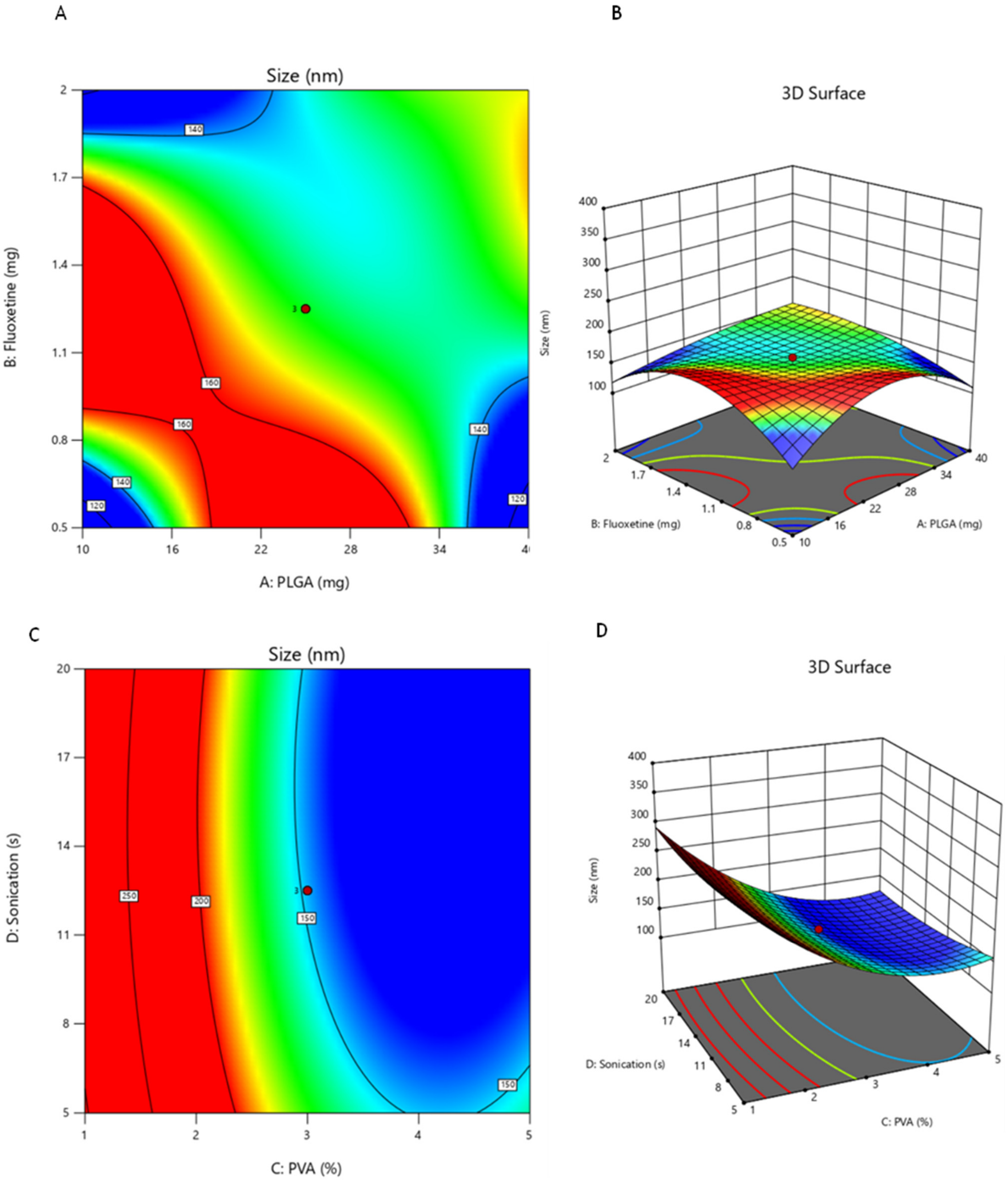

Figure S1. Contour plots (A and C) and surface plots (B and D) illustrating the effect of the independent variables on the NPs' size. The graphs are represented by color gradients, with bluer tones representing lower values and redder tones representing higher values.

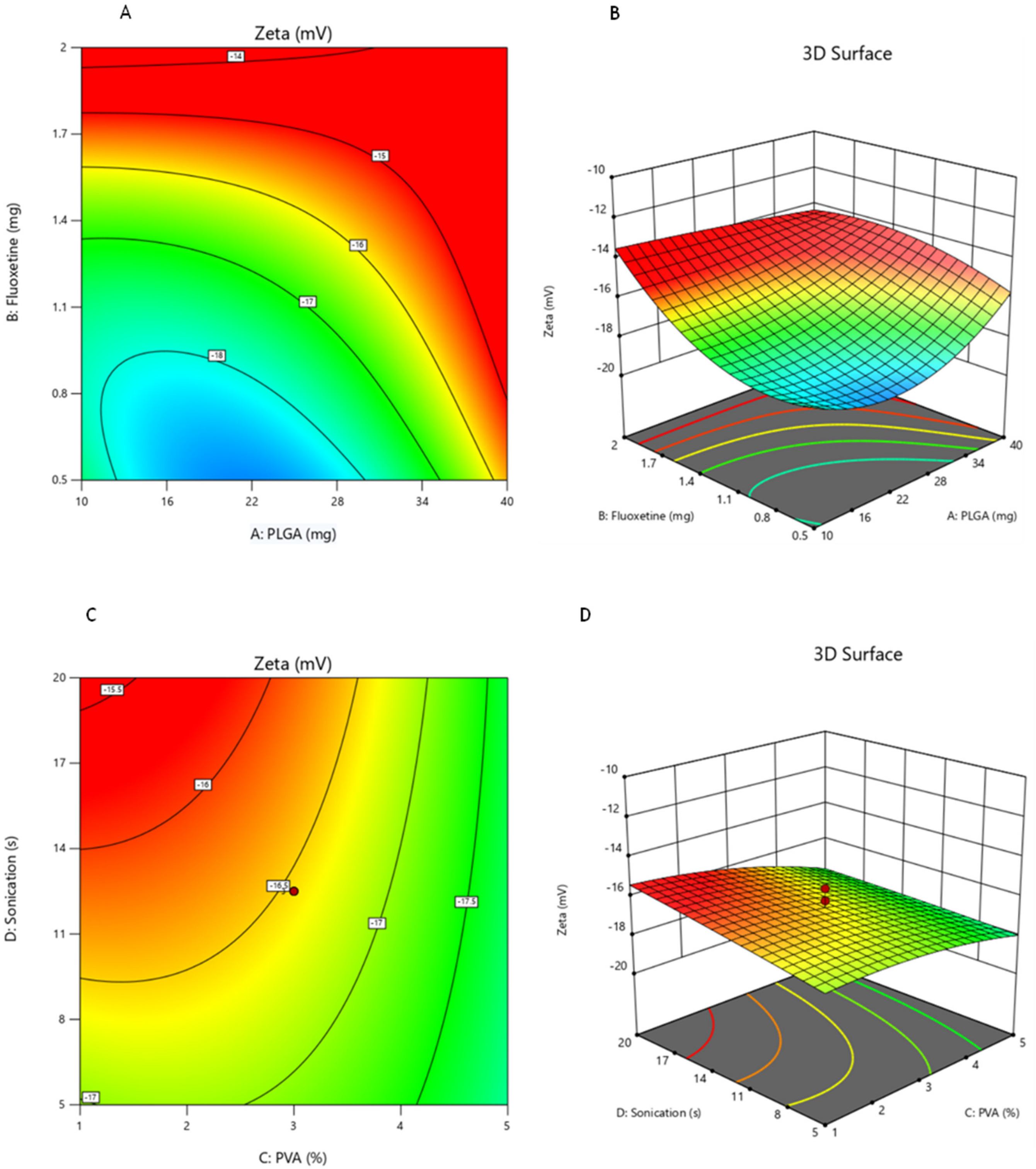

Figure S2. Contour plots (A and C) and surface plots (B and D) illustrating the effect of the independent variables on the NPs' zeta potential. The graphs are represented by color gradients, with bluer tones representing lower values and redder tones representing higher values.

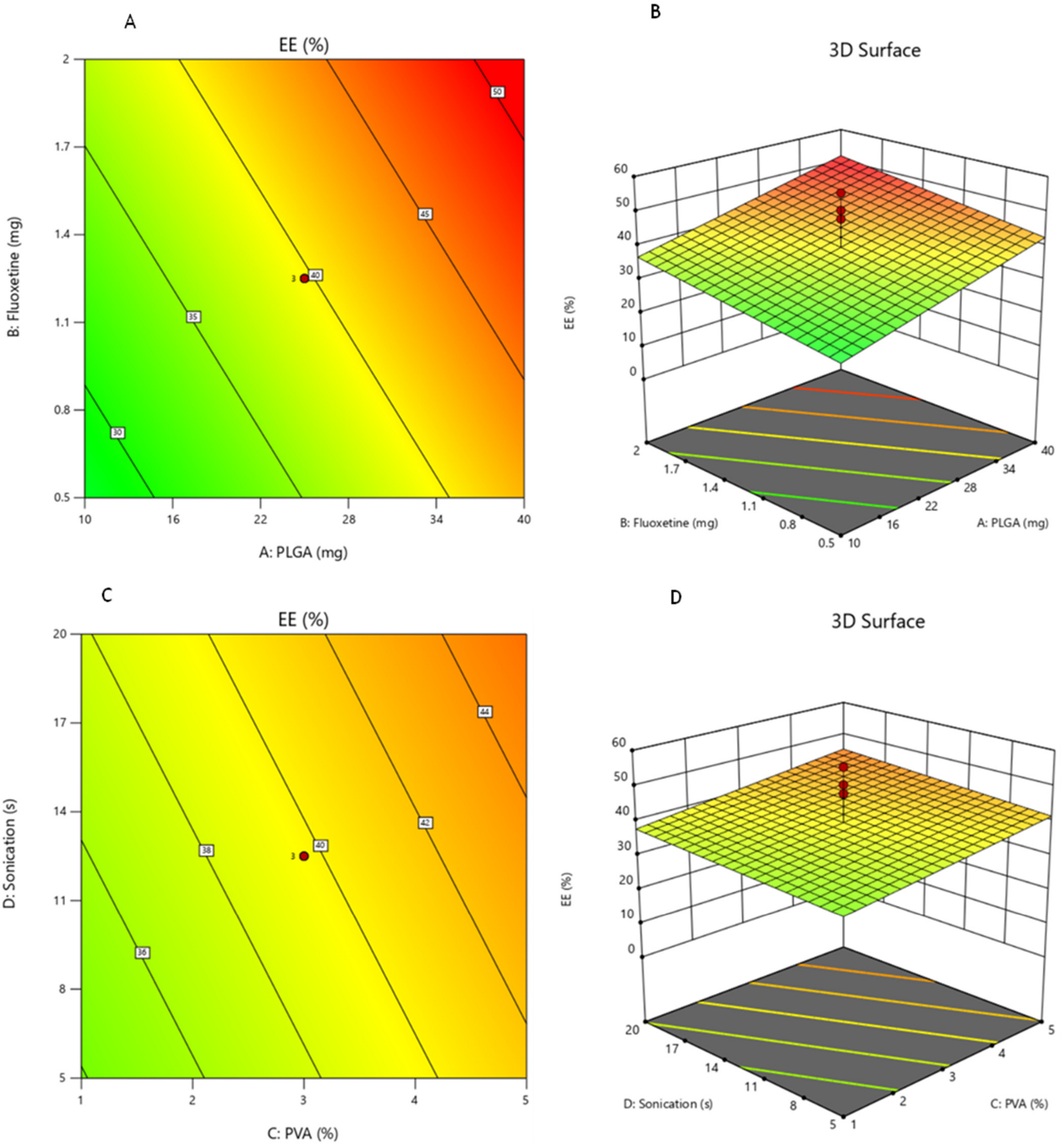

Figure S3. Contour plots (A and C) and surface plots (B and D) illustrating the effect of the independent variables on the NPs' EE. The graphs are represented by color gradients, with bluer tones representing lower values and redder tones representing higher values.

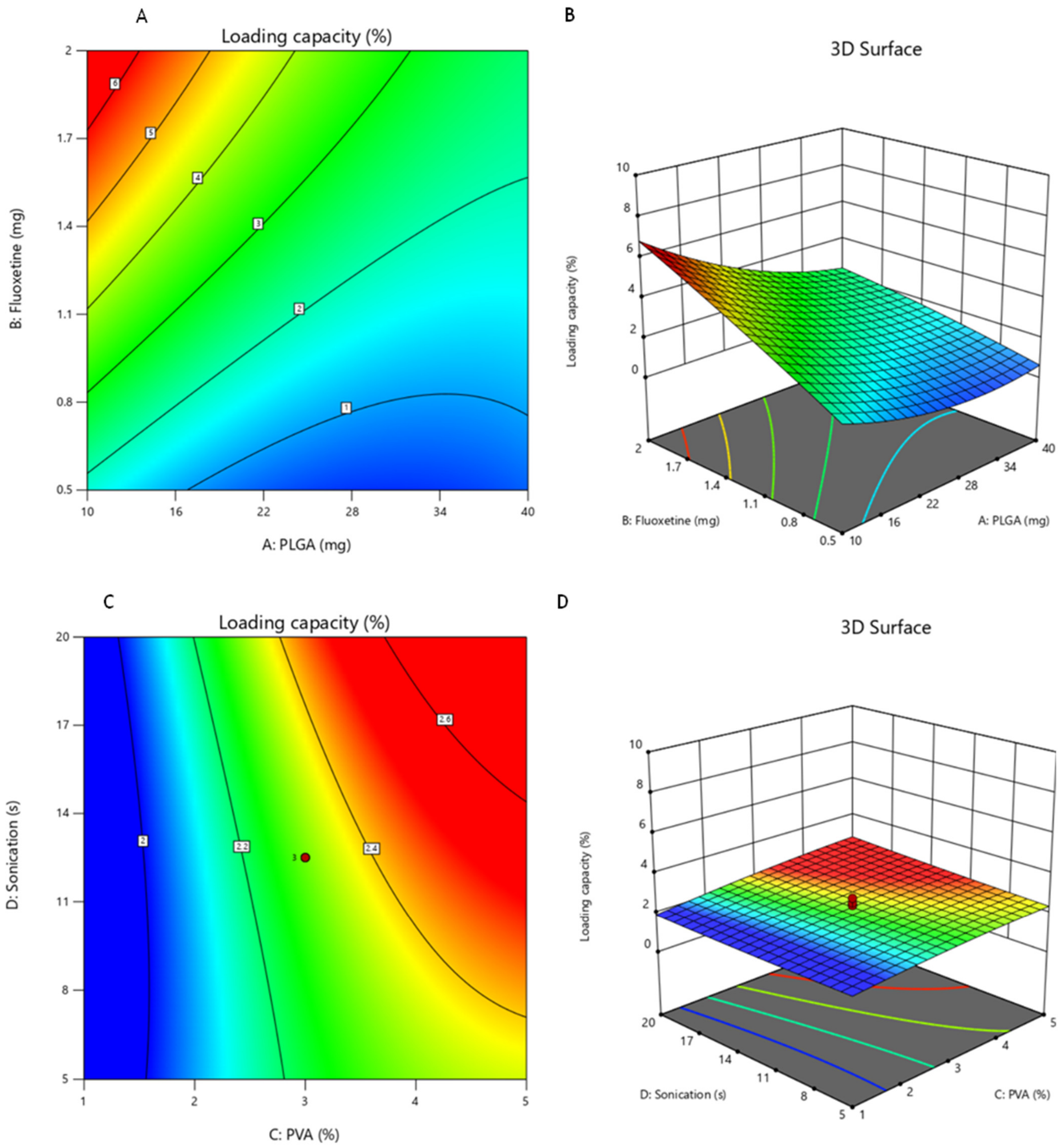

Figure S4. Contour plots (A and C) and surface plots (B and D) illustrating the effect of the independent variables on the NPs' LC. The graphs are represented by color gradients, with bluer tones representing lower values and redder tones representing higher values.

B. NPs' colloidal stability at storage conditions

Table S7. Evaluation of the colloidal stability of the FL-loaded PLGA NPs in storage conditions over 10 weeks. The physicochemical characterization was assessed by DLS, and the results are presented as mean and standard deviation (n=3). No significant changes were verified over time for size and PdI values ($p>0.05$), demonstrating that the NPs were stable for at least 10 weeks. The changes verified for zeta potential values ($p<0.05$) can be justified by the application of an electric field to the sample that can lead to modifications.

Time (weeks)	Size (nm)	PdI	Zeta potential (mv)
**0**	136 ± 2	0.22 ± 0.04	-14.6 ± 1.4
1	145 ± 9	0.27 ± 0.05	-13.3 ± 0.6
2	137 ± 3	0.21 ± 0.03	-13.6 ± 0.3
3	137 ± 3	0.25 ± 0.04	-11.8 ± 1.2
6	147 ± 5	0.28 ± 0.05	-10.4 ± 2.7
8	131 ± 5	0.21 ± 0.04	-11.2 ± 2.6
10	134 ± 3	0.20 ± 0.07	-10.0 ± 1.0

Table S8. Evaluation of the colloidal stability of the FA-FL-loaded PLGA NPs in storage conditions over 10 weeks. The physicochemical characterization was assessed by DLS, and the results are presented as mean and standard deviation (n=3). No significant changes were verified over time ($p>0.05$), demonstrating that the NPs were stable for at least 10 weeks.

Time (weeks)	Size (nm)	PdI	Zeta potential (mv)
**0**	167 ± 8	0.23 ± 0.07	-22.2 ± 0.3
1	168 ± 6	0.25 ± 0.07	-22.5 ± 2.3
2	174 ± 7	0.19 ± 0.06	-15.6 ± 7.8
3	170 ± 11	0.24 ± 0.05	-13.9 ± 2.1
6	164 ± 17	0.24 ± 0.05	-16.1 ± 2.9
8	167 ± 13	0.25 ± 0.08	-14.9 ± 6.2
10	166 ± 7	0.22 ± 0.09	-16.8 ± 5.2

C. FTIR spectrum

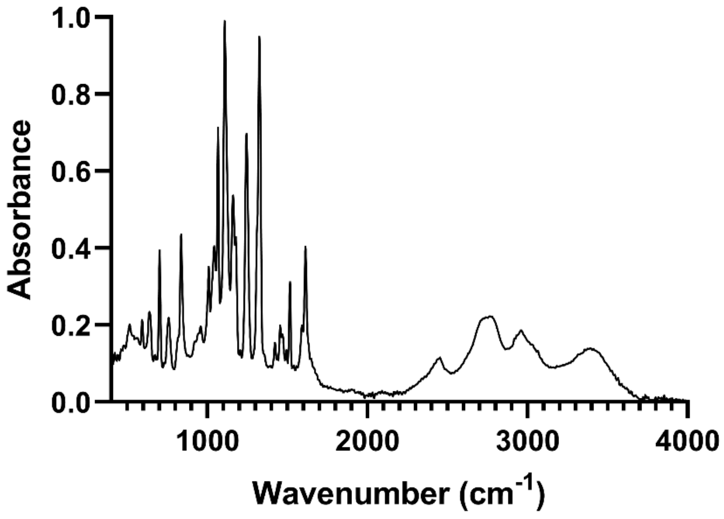

Figure S5. FTIR spectrum of free FL.

D. NPs' colloidal stability in simulated *in vitro* oral administration path

Table S9. Evaluation of the colloidal stability of the FL-loaded PLGA NPs in simulated digestion conditions. The physicochemical characterization was assessed by DLS, and the results are presented as mean and standard deviation (n=3). Changes in the zeta potential were observed, attributed to the protonation of the carboxyl groups in PLGA, which occurs both due to the acidic pH of the gastrointestinal media and the ionic composition of PBS.

Timepoint	Size (nm)	PdI	Zeta potential (mv)
0	142 ± 11	0.25 ± 0.02	-13.9 ± 3.0
After 2 h in gastric media	139 ± 5	0.21 ± 0.06	-0.3 ± 0.2
After 3 h in intestinal media	141 ± 3	0.21 ± 0.02	-6.0 ± 1.5
After 1 day in PBS	125 ± 7	0.23 ± 0.03	-9.5 ± 0.9
After 2 days in PBS	124 ± 4	0.29 ± 0.07	-9.5 ± 1.3
After 3 days in PBS	122 ± 1	0.21 ± 0.02	-9.1 ± 1.1
After 17 days in PBS	116 ± 7	0.24 ± 0.07	-8.1 ± 1.1

Table S10. Evaluation of the colloidal stability of the FA-FL-loaded PLGA NPs in simulated digestion conditions. The physicochemical characterization was assessed by DLS, and the results are presented as mean and standard deviation (n=3). Changes in the zeta potential were observed, attributed to the protonation of the carboxyl groups in PLGA, which occurs both due to the acidic pH of the gastrointestinal media and the ionic composition of PBS.

Timepoint	Size (nm)	PdI	Zeta potential (mv)
0	166 ± 8	0.21 ± 0.01	-21.9 ± 1,1
After 2 h in gastric media	171 ± 3	0.24 ± 0.04	-0.4 ± 0.6
After 3 h in intestinal media	172 ± 5	0.29 ± 0.13	-5.9 ± 1.4
After 1 day in PBS	172 ± 1	0.21 ± 0.05	-10.1 ± 0.8
After 2 days in PBS	171 ± 3	0.27 ± 0.06	-9.7 ± 0.4
After 3 days in PBS	170 ± 4	0.26 ± 0.05	-10.1 ± 2.3
After 17 days in PBS	168 ± 11	0.28 ± 0.09	-8.9 ± 1.0

E. Release kinetics parameters

Table S11. Release parameters for FA-conjugated and non-conjugated FL-loaded PLGA NPs obtained by fitting experimental data to different models for drug release kinetics.

	**FL-NPS**		**FA-FL-NPs**	
**Model**	**k**	**R^2**	**k**	**R^2**
Zero order	0.114	0.097	0.106	0.185
First order	0.006	3.610×10^{-4}	0.007	0.001
Korsmeyer-Peppas model	8.150	0.878	5.656	0.858
Hixson-Crowell model	3.264	0.029	3.264	0.029
Higuchi model	10.135	0.100	5.756	0.460

F. Cell studies

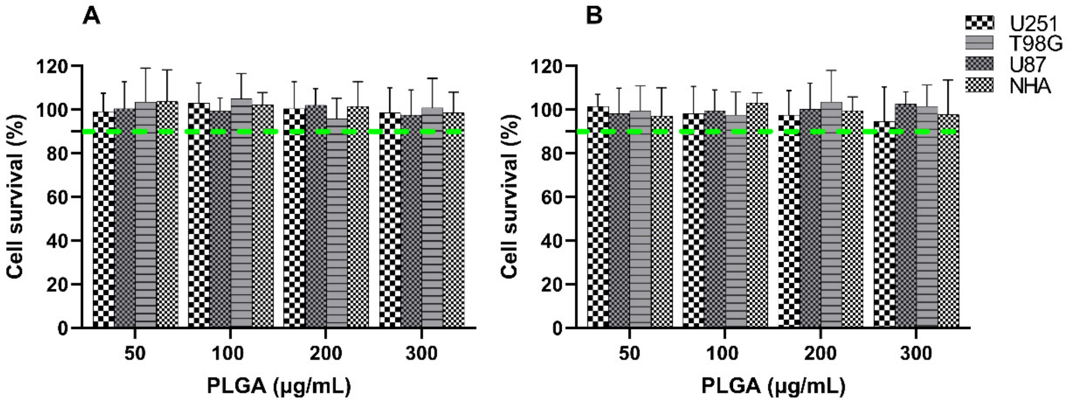

Figure S6. Effect of control unloaded (A) non-conjugated and (B) FA-conjugated PLGA NPs on the cell survival of U251, U87, T98g, and NHA cells, determined by SRB assay after 72 hours of treatment. Cell survival is presented as percent (%=T/C x 100). Data is given as mean ± SD (n = 3).
